# DNA Conserved in Diverse Animals Since the Precambrian Controls Genes for Embryonic Development

**DOI:** 10.1093/molbev/msad275

**Published:** 2023-12-12

**Authors:** Martin C Frith, Shengliang Ni

**Affiliations:** Artificial Intelligence Research Center, AIST, Tokyo, Japan; Graduate School of Frontier Sciences, University of Tokyo, Chiba, Japan; Computational Bio Big Data Open Innovation Laboratory, AIST, Tokyo, Japan; Graduate School of Frontier Sciences, University of Tokyo, Chiba, Japan

**Keywords:** evo-devo, homology, transcription

## Abstract

DNA that controls gene expression (e.g. enhancers, promoters) has seemed almost never to be conserved between distantly related animals, like vertebrates and arthropods. This is mysterious, because development of such animals is partly organized by homologous genes with similar complex expression patterns, termed “deep homology.” Here, we report 25 regulatory DNA segments conserved across bilaterian animals, of which 7 are also conserved in cnidaria (coral and sea anemone). They control developmental genes (e.g. *Nr2f*, *Ptch*, *Rfx1/3*, *Sall*, *Smad6*, *Sp5*, *Tbx2/3*), including six homeobox genes: *Gsx*, *Hmx*, *Meis*, *Msx*, *Six1/2*, and *Zfhx3/4*. The segments contain perfectly or near-perfectly conserved CCAAT boxes, E-boxes, and other sequences recognized by regulatory proteins. More such DNA conservation will surely be found soon, as more genomes are published and sequence comparison is optimized. This reveals a control system for animal development conserved since the Precambrian.

## Introduction

Genes often remain similar across vast gulfs of evolution. For example, the genes that encode ribosomal RNA (rRNA) in humans and bacteria have recognizably similar DNA sequences. Gene expression is controlled by DNA segments near the genes, which curiously lack such long-term conservation. Some are conserved across vertebrate animals, but few have been found conserved between vertebrates and invertebrates (Vavouri et al. 2007; Harmston et al. 2013; Maeso et al. 2013).

**Fig. 1.**
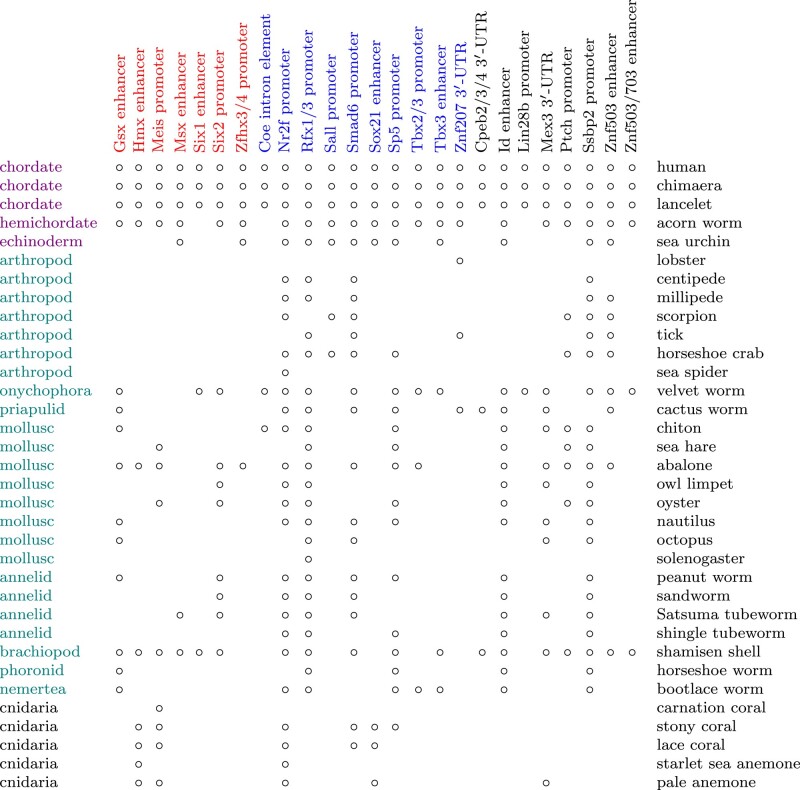
Animals in which each regulatory DNA element was detected. Regulatory segments are colored according to their nearby gene: homeobox genes red (left), other transcription factors blue (middle). Each animal’s phylum is written on the left, deuterostomes in purple (top) and protostomes in green (middle). The animals’ scientific names are in supplementary Table S1, Supplementary Material online.

Early animal evolution produced bilaterian animals, which then split into two superphyla: protostomes and deuterostomes (Fig. 1). A pioneering study by Royo et al. (2011) found several regulatory DNA segments conserved across deuterostomes, but found none of them in any nondeuterostome, except, amazingly, two in sea anemone, which is not even bilaterian. These two are enhancers of the *Sox21* and *Hmx* genes. Soon after, Clarke et al. (2012) found two regulatory elements conserved between deuterostomes and protostomes: an enhancer of the *Id* gene in gastropods (but no other protostomes), and an enhancer of *Znf503* in tick (but no other protostomes). Beyond this handful of exceptions, it seems that gene-regulating DNA is not conserved across bilaterians, suggesting that their developmental programs are not conserved either.

Regulatory DNA conserved across vertebrates or beyond tends to regulate genes that control embryonic development (Maeso et al. 2013). These genes often encode transcription factors: proteins that control other genes by interacting with their regulatory DNA. One family of transcription factor genes contain homeobox sequences, which encode a DNA-binding structure called the homeodomain: these are especially used in development. Development involves the appearance of different territories in the embryo that express different combinations of transcription factors, and then develop into different body parts. These territories form in response to varying concentrations of signaling molecules, such as BMP, Wnt, and hedgehog proteins, that are secreted from cells. Often, one gene contributes to many parts of development, presumably because development evolved by redeploying these genes.

It is curious that developmental genes are widely conserved across animals, but their regulatory DNA rarely is. One theory is that conserved regulatory segments correspond to conserved body plans such as vertebrate (Vavouri et al. 2007). This does not explain, however, why the expression patterns of developmental genes are often deeply conserved across animals (Maeso et al. 2013). One possible answer is that regulatory function can be conserved even as the DNA evolves so much that sequence similarity is lost (Wong et al. 2020).

Developmental genes often have multiple enhancers that are conserved across vertebrates. The homologous genes in flies seem to have multiple enhancers that are conserved in flies, though none resemble the vertebrate enhancers. The same trend is found in nematode worms (Vavouri et al. 2007) and sea urchins (Tan et al. 2019). For example, the *Meis2* gene has the largest number of nonprotein-coding DNA segments conserved in vertebrates, its fly homolog the most segments conserved in flies, and its sea urchin homolog the most segments conserved in sea urchins (Tan et al. 2019). One explanation could be independent evolution by redeploying developmental genes. Another idea is that these genes had multiple conserved enhancers in ancestral bilaterians, and the enhancers slowly “turned over,” i.e. new ones appeared and old ones disappeared while perhaps retaining the old function (Harmston et al. 2013).

Here, we find many more DNA segments that control developmental gene expression and are conserved across bilaterian animals, and even in nonbilaterian corals and sea anemone. They were found by exploiting the wealth of recently published animal genomes, and by optimizing DNA homology detection (Frith 2023). This reveals a system of DNA sequences conserved since the Precambrian that control animal development.

## Results and Discussion

The first step was to find conserved DNA segments between the genomes of human and chimaera (*Callorhinchus milii*), a cartilaginous fish. These genomes are related closely enough that many conserved segments can be found, but distantly enough that most of their DNA lacks similarity. Another reason for using chimaera is that its genome has evolved slowly (Venkatesh et al. 2014). Next, conserved regions were removed if they encode protein, rRNA, tRNA, snRNA, snoRNA, or miRNA. Pseudogenes were also removed, i.e. DNA that used to encode protein, rRNA, tRNA, etc. (Such DNA might also regulate gene expression, but its conservation can be explained by what it does or did encode.) The remaining conserved regions cover 5.7 million base-pairs in chimaera: 1/174-th of the genome.

The next step was to seek conserved segments between these chimaera regions and various invertebrate genomes. By not using the whole chimaera genome, we get 174-fold fewer false matches, so we can find 174-fold weaker similarities. Each similarity was given an *E*-value, which means the number of times such a similarity would be expected between random sequences of shuffled bases. More specifically, between random sequences with 40% g+c and the same lengths as: the one invertebrate genome, and all the chimaera regions. Similarities were accepted with either *E*-value ≤0.0001 or *E*-value ≤10 and near homologous genes. For example, a chimaera segment near the *smad6* gene matched octopus DNA with *E*-value 0.46, and the nearest gene in octopus is the homolog of *smad6*.

As a negative control, each search was repeated after reversing (but not complementing) the invertebrate genome. DNA does not evolve by reversal, because that would require flipping the $3^{\prime }$-to- $5^{\prime }$ bonds between all the nucleotides. In most cases a reversed genome had about 10 matches with *E*-value ≤10, and the lowest *E*-value ever seen was 0.0009.

Further sensitivity was gained by using conserved parts of invertebrate genomes. For example, by using oyster DNA segments that are conserved in scallop (1/59-th of the oyster genome), sensitivity was boosted a further 59-fold, revealing matches near the *Ptch* and *Meis* genes.

**Fig. 2.**
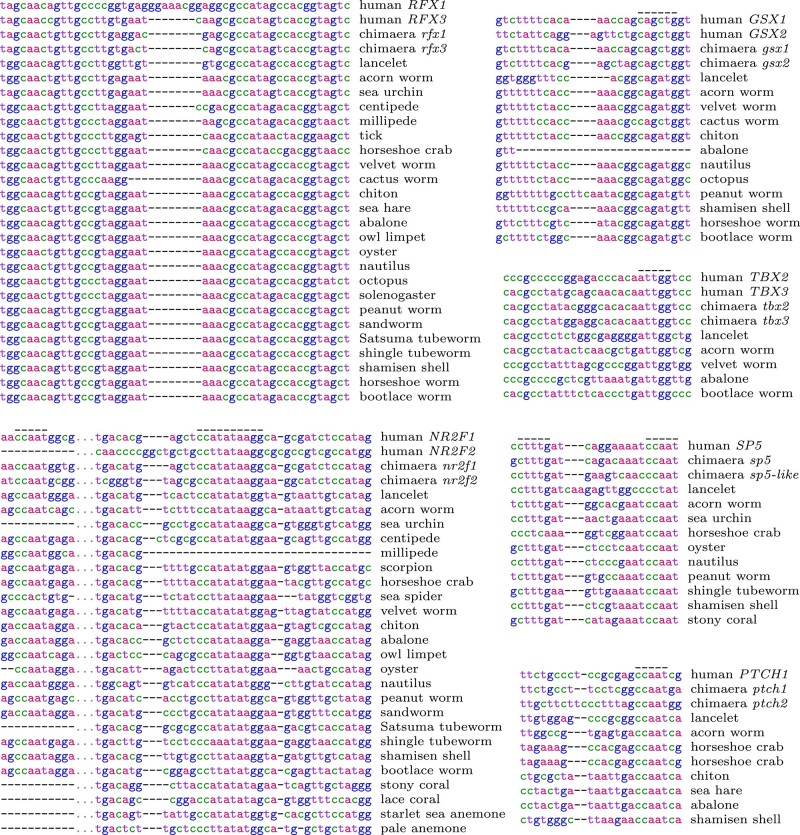
Parts of alignments between regulatory DNA sequences. The full alignments are in the Supplement. The over-bars show: CCAAT boxes (in the *Nr2f*, *Sp5*, and *Ptch* promoters), reverse-strand CCAAT box ATTGG (*Tbx2/3* promoter), E-box CAnnTG (*Gsx* enhancer), Wnt response element CTTTG (*Sp5* promoter), and CArG box CC(A/T)${}_{6}$GG (*Nr2f* promoter). The *Ptch* promoter matches 2 places in the horseshoe crab genome, both near genes annotated as “patched-like.”

**Fig. 3.**
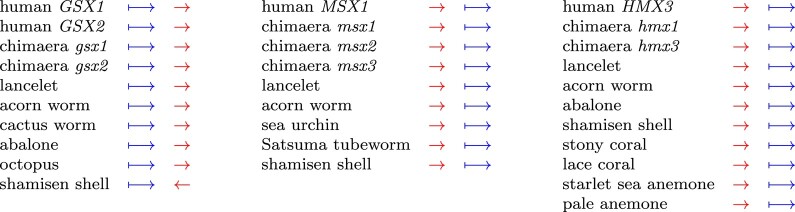
Position and orientation of conserved DNA segments (red arrows) relative to nearby genes (blue arrows with bars). Within each column, all the genes are homologous to each other. Animals without gene annotations are not shown. A regulatory segment does not really have an orientation, but once an orientation is arbitrarily chosen for any one segment, that defines the orientations of all its homologs.

These methods found 25 DNA segments with similarity between vertebrates and nondeuterostome animals (Fig. 1). They can be explained by either common ancestry or convergent evolution. It seems implausible that most are due to convergent evolution, because they are in a wide range of animals (Figs. 1 and 2), and they preserve position and orientation relative to the genes (Fig. 3). The segments were not found in some invertebrates (Fig. 1): either they were lost during evolution of those animals, or they are present but undetected. The segments are labeled “promoter” if they occur at a gene’s transcription start site in human (the best-annotated genome), else “enhancer.” One segment lies in an intron, and three lie in 3${}^{\prime }$-UTRs (UnTranslated Regions of exons, downstream of the translated protein-coding region).

Interestingly, all of these genes, except *Znf207*, have multiple (2–4) copies in genomes of jawed vertebrates. For example, jawed vertebrates have 3 similar *Meis* genes (*Meis1*, *Meis2*, and *Meis3*), 2 *SoxB2* genes (called *Sox14* and *Sox21*), 2 *sine oculis* genes (*Six1* and *Six2*), and 2 *NET* genes (*Znf503* and *Znf703*). *Sp5* is a semiexception: vertebrates have 2 copies, named *Sp5* and *Sp5-like*, but *Sp5-like* was lost in mammals and birds (Pei and Grishin 2015). In early vertebrate evolution, there were 2 rounds of whole-genome duplication. Most gene duplicates were rapidly lost, so that vertebrates do not have many more genes than invertebrates, but some genes remain in 2–4 copies. These retained multicopy genes tend to be developmental (Blomme et al. 2006), but it still seems remarkable that they include nearly all the genes found here. One explanation could be that genes with many enhancers and roles are more likely to partition their functions after duplication (Force et al. 1999).

The conserved DNA segments were detected at all, some, or just 1 of these multicopy genes. For example, the conserved *Gsx* enhancer was found near both *Gsx* genes in human and chimaera (Figs. 2 and 3), but the *SoxB2* enhancer was only found at *Sox21*. The *sine oculis* enhancer was only found at *Six1*, whereas the *sine oculis* promoter was only found at *Six2*. One *NET* enhancer was found at both *Znf503* and *Znf703*, the other only at *Znf503*. While “not found” does not necessarily mean “absent,” it seems that duplication of these genes enabled divergence of their regulation and developmental roles.

The functions of these genes and conserved DNA segments are partially known. *Gsx* and *Msx* encode transcription factors that define territories in early development of the central nervous system (CNS), in a manner conserved across bilaterian animals (Tomer et al. 2010; Arendt et al. 2016; De Robertis and Tejeda-Muñoz 2022). They produce an ancient sensory/inter/motor neuron system. *Msx* is expressed at the lateral edges of the CNS, and produces Rohon-Beard mechanosensory neurons. *Gsx* is expressed more medially and produces interneurons, and the most medial territory produces motor neurons. The *Gsx* enhancer seems to contribute by preventing lateral expression of *Gsx*, thus defining the brain’s pallium–subpallium boundary (Desmaris et al. 2018; Salomone et al. 2021). *Msx* plays many other roles in development: the enhancer is the previously identified *Msx1* “proximal element” that produces expression in mouse embryo nasal epithelium, myotome, limb mesenchyme, eye, ear, roof plate, second arch, genital ridge and epiphysis (MacKenzie et al. 1997).

The *Six1/2* genes control development of eyes and other cranial sensory organs (e.g. olfactory epithelium, inner ear, taste papillae), and also contribute to development of kidney, thymus, parathyroid gland, and more (Sato et al. 2012). The *Six1* enhancer is the previously described Six1-13 element, which produced variable expression patterns of unclear importance (Sato et al. 2012): it would be interesting to reexamine this element.

*Tbx2/3* control formation of the brain’s neurohypophysis (Trowe et al. 2013) and contribute to development of vertebrate limbs, heart, liver, and more (Papaioannou 2014). The *Tbx3* enhancer was shown to affect expression of *Tbx3* and limb length in mice and horses (Liu et al. 2022).

The *Coe* genes encode transcription factors affecting subpallium development, expressed in *Gsx*+ brain regions of mice and annelids (Tomer et al. 2010). They also define subtypes of body-wall motor neuron in mice and roundworms (Catela et al. 2019) and promote development of fat cells (Wang et al. 2017). The conserved DNA segment is in an intron, so is transcribed into RNA: it might function in the DNA or RNA or both. This RNA region, of the mouse *Coe* gene *EBF1*, interacts with PSPC1 protein during fat cell development, promoting export of the RNA from the nucleus and expression of EBF1 protein (Wang et al. 2017).

The *Znf503/703* genes encode transcriptional repressors that are related to Sp transcription factors, but are thought not to bind directly to DNA. They contribute to brain and eye development in vertebrates and flies. In vertebrates they define hindbrain territories and motor neuron subtypes, and contribute to development of the brain’s striatum, and limbs (Pereira et al. 2016). The *Znf503* enhancer was previously found in tick (Clarke et al. 2012): here it is found in diverse protostomes (Fig. 1). The *Znf503/703* enhancer is 2.5 kb further upstream: it directs expression (in mouse embryos) in branchial arches and their derivatives, apical ectodermal ridge of limb buds, and urogenital tissues (Chang et al. 2013).

The other genes also regulate development (Lorente-Sorolla et al. 2018; Robinton et al. 2019; Guntur et al. 2021; Parker et al. 2021). *Hmx* interacts with *Gsx* in development of the brain’s hypothalamus (Wang et al. 2004). *Ptch* genes encode the main receptors that recognize hedgehog signaling molecules, and Smad6 proteins inhibit response to BMP signaling molecules (Miyazawa and Miyazono 2017). *Sp5* is a target of Wnt signaling in bilaterians, and also in cnidaria where it prevents development of multiple heads (Vogg et al. 2019). It has a promoter region conserved from human to coral, which includes an especially conserved sequence resembling a Wnt response element (Fig. 2). The *Rfx1/3* genes control formation of cilia on cells, which are used for hedgehog signaling, establish left–right body asymmetry, and more (Choksi et al. 2014). The promoter is spectacularly conserved across bilateria (Fig. 2). Ssbp2 protein partners with LIM homeodomain proteins, which specify motor neurons in the same way in vertebrates and flies, among other roles (Yasuoka and Taira 2021). *Nr2f* participates in neural development of bilaterians and cnidaria (Gauchat et al. 2004): its promoter has been conserved since early neural evolution (Figs. 1 and 2).

Interestingly, the three genes with conserved 3${}^{\prime }$-UTR segments encode proteins that bind to RNA. The Cpeb proteins control mRNA localization and translation, in neural development and memory formation (Kozlov et al. 2021). The *Cpeb* mRNAs are regulated by miRNAs binding to their 3${}^{\prime }$-UTRs (Morgan et al. 2010; Vetere et al. 2014; Margvelani et al. 2018). *Mex3* contributes to defining anterior territories in bilaterian embryos, by destabilizing mRNAs (Takada et al. 2009; Yang et al. 2017). Its 3${}^{\prime }$-UTR contains elements for translational enhancement and destabilization, including by its own protein or miRNA (Takada et al. 2009; Umehara et al. 2020). The UTR was previously found conserved between vertebrates and lancelet (Takada et al. 2009): here it is found in other phyla and even a sea anemone (Fig. 1). The Znf207 protein is a bit of an outlier: it contributes to chromosome alignment during cell division, a function not specific to animal development. It also, however, contributes to early neural development by binding to DNA or RNA (Malla et al. 2022).

Do these particular developmental genes have a common theme? It is hard to say, but perhaps there is a tendency for roles in ventral CNS (e.g. subpallium, striatum, hypothalamus, neurohypophysis, motor neurons).

We also examined human genome-wide predictions of which enhancers regulate which genes (Nasser et al. 2021). Their DNA elements overlap all but one (*GSX1*) of our enhancers, none of our seven 3${}^{\prime }$-UTR segments, and one (*EBF3*) out of three intron segments. This suggests that the UTR segments are not enhancers: their conservation may reflect RNA function. Each enhancer is predicted to regulate the expected gene, for example the *SIX1* enhancer is predicted to regulate *SIX1*. However, the enhancers are often predicted to regulate more than one gene. It seems these other interactions are not always conserved: e.g. the *HMX3* enhancer is predicted to regulate *BUB3*, which is 20 kb away, but the lancelet homologs are separated by 15 million bp. It would be interesting to have similar predictions for other animals, to see the evolution of enhancer-gene links.

In the conserved DNA segments, conserved transcription factor binding sites were identified (see the Supplementary material online), using the JASPAR database of DNA-binding tendencies (Castro-Mondragon et al. 2022). The results are uncertain, because transcription factors have weak sequence preferences, often similar to those of other transcription factors. Perhaps different factors bind to overlapping sites at different times depending on molecule concentrations.

On the other hand, there are clearly conserved “boxes,” including E-boxes and many CCAAT boxes (Fig. 2). The E-box, CAnnTG, binds to various transcription factors that have a bHLH (basic-helix-loop-helix) structure. bHLH factors initiate neural development in bilateria and cnidaria (Arendt et al. 2016), perhaps by binding to these conserved E-boxes. CCAAT boxes bind to the transcription factor NF-Y, which controls many developmental genes (Stanney et al. 2020). One CArG box was found, conserved from human to sea anemone (though imperfect in coral), in the *Nr2f* promoter (Fig. 2). In general, CArG boxes bind to the Srf transcription factor, which controls cell migration and neural outgrowth in development (Miano 2010).

Another interesting result is that the conserved DNA segments rarely change position (upstream/downstream) or orientation relative to the nearby genes. Fig. 3 shows just 1 change, for *Gsx* in shamisen shell. There are a few others. In chimaera, the *sox21* gene has been inverted relative to its neighboring genes, but the inverted region does not include the enhancer. So, the enhancer is upstream of *sox21* in chimaera but downstream in the other animals.

Most of these genes lie in clusters of nonprotein-coding DNA segments conserved between human and chimaera (supplementary Fig. S2, Supplementary Material online). However, some have few such segments (supplementary Figs. S3–S9, Supplementary Material online), including the genes that encode RNA-binding proteins (*Cpeb2/3/4*, *Lin28b*, *Mex3*, *Znf207*). They usually lie in clusters of human-chicken conserved segments, but *Rfx1* does not. This further suggests there is something special and different about the uber-conserved *Rfx1/3* promoter.

Some protostome animals have more detected DNA elements than others (Fig. 1). This is partly artifactual, e.g. some genomes lacked gene annotations so had the stricter 0.0001 *E*-value threshold. With that said, these seem to have many conserved elements: abalone, shamisen shell, and velvet worm (which lacks gene annotations). Perhaps these animals have undergone less evolution of their development and body plan. All 3 have sometimes been regarded as “primitive” or “living fossils”: the shamisen shell (*Lingula*) was noted in Darwin’s Origin of Species as little changed from ancient fossils.

On the other hand, no conserved segments were found in fly (*Drosophila melanogaster*), roundworm (*Caenorhabditis elegans*), or leech (*Helobdella robusta*). This might be because their lineages evolved rapidly, including their regulatory DNA which diverged or turned over faster. It is also possible that their developmental systems have evolved more drastically, for example how BMP defines dorsal and ventral territories in *Helobdella* (Kuo and Weisblat 2011).

Beyond bilateria, no conserved segments were found in ctenophores (comb jellies), sponges, or *Trichoplax* (supplementary Table S2, Supplementary Material online). A recent study found 3 regulatory DNA elements conserved between mammals and flies (Bridi et al. 2020): we confirmed they are conserved between human and chimaera, but did not find them in invertebrates. Even when comparing each pair of human and fly sequences, boosting sensitivity $10^{12}$-fold over comparing the whole genomes, our methods found tiny similar fragments in just 1 out of 3 pairs. Another study found identical DNA segments ≥50 bp between human and fly, and even human and sponge (Ryu et al. 2012). We confirmed that such intriguing matches exist, though they overlap regions that encode protein, rRNA, tRNA, or snRNA, or pseudogenes. A third study found 6,533 nonprotein-coding segments conserved across deuterostomes (Simakov et al. 2015), vastly more than found here or elsewhere (Royo et al. 2011; Clarke et al. 2012). Some possible reasons are that they used *E*-value ≤0.05 per 5 kb (which is 1 per 100 kb), and did not exclude pseudogenes or RNA genes.

In summary, we begin to see a system of DNA elements controlling gene expression for animal development, conserved since ancestral bilaterians and beyond in the Precambrian. This conservation helps us understand how early animals developed, and how modern development is built on these ancient components.

These bilaterian-conserved DNA segments are strikingly fewer than the thousands conserved across jawed vertebrates, even though the last common ancestor of jawed vertebrates (∼460 million years ago) is more than halfway to the last common ancestor of bilaterians (∼600–700 million years ago). In contrast, gene-regulating DNA is rarely conserved between *Drosophila* and mosquitos (Engström et al. 2007), even though both are flies (Diptera). This suggests there was a “big freeze” of developmental enhancers in early vertebrates, perhaps reflecting a step change in developmental intricacy. It will be interesting to survey conservation of such DNA segments in diverse groups of animals.

It is not yet clear why some regulatory elements are more conserved than others. One trend is that the bilaterian-conserved elements are enriched for promoters (12 out of 25) versus enhancers. Perhaps these promoters are more exquisitely constrained because they integrate regulatory inputs to the gene. Another possibility is that these DNA segments are transcribed into RNA and conservation reflects RNA function.

Why have these DNA sequences been so exceptionally conserved? Judging by present-day bilaterians and cnidaria, the common ancestor of these animals already had an exquisitely integrated multicellular body, with a nervous system, that developed from a single fertilized cell (Arendt et al. 2016; De Robertis and Tejeda-Muñoz 2022). This intricate development must have evolved by accretion of developmental processes, often by redeploying existing pathways (Wang et al. 2004). So, 1 regulatory DNA element could become used in many parts of development, or its outcomes might get built upon by many processes. This brings us to Hyrum’s Law of software development: “With a sufficient number of users…all observable behaviors of your system will be depended on by somebody” (Wright 2017). Thus, DNA changes with even subtle effects on transcription factor interactions, e.g. though DNA shape (Gordân et al. 2013), would likely be harmful. This resulted in strongly conserved DNA controlling core animal development, upon which evolved arthropods, molluscs, chordates, and so on.

## Supplementary Material

msad275_Supplementary_Data

## Data Availability

The alignments between pairs of whole genomes (e.g. chimaera–human, oyster–scallop) are in the 2023 section of https://github.com/mcfrith/last-genome-alignments. Other files used in this study are at https://github.com/mcfrith/conserved-animal-dna.
