# Supplementary material for: DNA Conserved in Diverse Animals Since the Precambrian Controls Genes for Embryonic Development
Source: Mol Biol Evol. 2023 Dec 12;40(12):msad275. doi: 10.1093/molbev/msad275 (PMC10735318; doi:10.1093/molbev/msad275)
Supplement: msad275_Supplementary_Data [file msad275_supplementary_data.pdf]

Supplement to: DNA conserved in diverse animals since the Precambrian controls genes for embryonic development

Martin C. Frith[1,2,3] and Shengliang Ni[2]

[1]Artificial Intelligence Research Center, AIST
[2]Graduate School of Frontier Sciences, University of Tokyo
[3]Computational Bio Big Data Open Innovation Laboratory, AIST

Methods

Finding conserved segments between the human and chimaera genomes

The genomes were compared using LAST version 1453 (https://gitlab.com/mcfrith/last). First, `chrEBV` (Epstein-Barr virus) was removed from the human genome. An index called (say) `cdb` was made for the chimaera genome:

```
lastdb -P8 -uMAM8 -c cdb chimaera.fa
```

`-P8` makes it faster by using 8 processors, `-uMAM8` increases sensitivity and memory use [1], and `-c` suppresses simple sequences like `atatatatatatatatat`. Next, rates of insertion, deletion, and substitutions between the genomes were found [2]:

```
last-train -P8 --revsym -D1e9 --sample-number=5000 cdb human.fa > hc.train
```

Then, many-to-one human-to-chimaera alignments were found (based on these rates) [3]:

```
lastal -P8 -D1e9 -m100 --split-f=MAF+ -p hc.train cdb human.fa > many-to-one.maf
```

`-m100` makes it more slow and sensitive, and `-D1e9` gets similarities that would be expected between shuffled-base sequences at most once per 10^9 human bp. The alignments were cut down to one-to-one alignments [3]:

```
last-split -r many-to-one.maf > one-to-one.maf
```

Finally, isolated alignments (not near other alignments) were discarded, using `maf-linked` from https://gitlab.com/mcfrith/protein-fossils/:

```
maf-linked one-to-one.maf > linked.maf
```

This final step removes non-homologous insertions of homologous transposons [4].

Human genes and pseudogenes

In order to find conserved regulatory DNA, we need to exclude conserved DNA in protein-coding regions, RNA genes, and pseudogenes. The following genome coordinates were used, where `.txt` and `.bigPsl` refer to files in the UCSC genome database:

- Protein-coding regions in `ncbiRefSeq.txt`, `wgEncodeGencodeCompV43.txt`, `xenoRefGene.txt`, and `hg38.refseq.transMapV5.bigPsl`.
- rRNA, tRNA, and snRNA genes and pseudogenes in `rmskOutCurrent.txt`.
- Exons of RNA genes in `ncbiRefSeq.txt`, extracted like this:

  ```
  awk '$13 ~ /^RNU|^MIR|^SNOR|^SCARNA|^RNA5-8/' ncbiRefSeq.txt
  ```

- Protein fossils derived from host genes, in https://github.com/mcfrith/protein-fossils.

Chimaera genes and pseudogenes

First, repeats were found with RepeatMasker 4.1.5 (using RMBlast 2.13.0 and TRF 4.09):

```
RepeatMasker -xsmall -engine rmblast -s -species "Callorhinchus milii" chimaera.fa
```

Then, protein fossils were found as described previously [4], except that `fasta-nr` option `-s` was used this time. Finally, these genome coordinates were obtained:

- Protein-coding regions and RNA genes in the genome's `genomic.gff` file, extracted like this:

  ```
  awk -F"\t" '$3 ~ /CDS|rRNA|tRNA|snRNA|snoRNA|guide_RNA/' genomic.gff
  ```

  Here, `guide_RNA` means scaRNA (small Cajal body-specific RNA), which is a type of snoRNA.

- rRNA, tRNA, and snRNA genes and pseudogenes from RepeatMasker.
- Protein fossils derived from host genes.

Non-genic chimaera DNA conserved in human

First, each human (pseudo)gene segment was extended by 50 base-pairs in both directions. This is because the edges of the human-to-chimaera alignments cannot be perfectly reliable: they may overshoot slightly beyond conserved regions into non-conserved DNA, or undershoot [5]. Then, chimaera bases were retained if they align to human bases not in these segments. These chimaera regions were extended by 50 bases in both directions. Finally, bases in chimaera (pseudo)genes were removed from these regions.

Comparing invertebrate genomes to these chimaera regions

An index called (say) `sdb` was made for the chimaera DNA regions:

```
lastdb -c -uMAM8 -S2 sdb chimaera-segments.fa
```

Then, similar segments were sought between each genome and the chimaera regions:

```
lastal -P8 -J1 -D$D -m100 -p my.train sdb genome.fa > out.maf
```

Here, `$D` was set to (genome size) / 10: this gets similarities that would be expected at most once per `$D` genome base-pairs if we compared a shuffled genome to shuffled DNA with the same length as the whole of `sdb`. In other words, it gets similarities with E-value ≤ 10. This step requires a file `my.train`, which is explained later.

E-value adjustment

For each pair of similar segments, `lastal` outputs a per-sequence E-value. This is the number of times such a similarity would be expected between random DNA sequences with the same lengths as: the one sequence from `genome.fa` (chromosome or fragment), and the whole of `sdb`. These E-values were converted to per-genome E-values:

genome E-value = (sequence E-value) $\times$ (genome length) / (sequence length) .

Simple sequences and reversed genomes

Simple sequences, like `atatatatatatatatat`, evolve frequently and independently. This leads to similar segments that are not conserved homologs. There are several methods to suppress simple sequences from homology search, but these methods do not work equally well. LAST uses the `tantan` method, which seems to suppress them effectively [6].

One way to test this is by comparing two DNA sequences after reversing (but not complementing) one of them. Then there are no true homologs, but simple sequences produce many strong similarities [6]. In the present study, reversed genomes did not have much stronger matches than would be expected between random sequences of shuffled bases, indicating that simple sequences were suppressed effectively.

Finding conserved regions between invertebrate genomes

This step found, for example, oyster DNA segments that are conserved in scallop. The genomes were compared in the same way as human-to-chimaera, without the final `maf-linked` step. Oyster protein-coding regions and RNA genes were extracted like this:

```
awk -F"\t" '$3 ~ /CDS|rRNA|tRNA|snRNA|snoRNA|guide_RNA/' genomic.gff
```

Each of these regions was extended by 50 base-pairs in both directions. Then, these regions were cut from the oyster segments that align to scallop. The remaining oyster segments were then extended by 50 bases in both directions.

This was repeated for the genome pairs in table S4, with oyster replaced by the left genome and scallop by the right genome. Some of these genomes did not have a `genomic.gff` file, so an empty file was used instead.

The `my.train` file

This file has rates of insertion, deletion and substitutions. It defines "similar": for example, it might specify that `a`↔`g` and `c`↔`t` substitutions are more likely than other substitutions.

Typically, `last-train` finds these rates from the sequences we wish to compare [2]. But that doesn't work here, because the invertebrate DNA is too distantly related to the chimaera regions.

Therefore, a more closely-related animal, lamprey (a jawless vertebrate), was used instead. Using the whole lamprey genome doesn't work well either, so lamprey DNA segments were used that are conserved in hagfish (another jawless vertebrate). These conserved lamprey segments were found in the same way as oyster-versus-scallop, removing protein-coding regions and RNA genes in the same way too. These lamprey segments were compared to chimaera in two ways:

```
last-train -s1 --revsym --matsym --gapsym -D1e7 --sample-number=5000
                                                            sdb lampreySegs.fa > try1.train

last-train -s1 --revsym --matsym --gapsym -D1e7 --sample-number=5000 --pid=65 --scale=6
                                                            sdb lampreySegs.fa > try2.train
```

`--pid=65` makes `last-train` ignore similarities with $>65\%$ identity, so the rates will be tuned for finding homologies with low percent-identity, but will be worse for finding short homologies with high percent-identity. (`--scale=6` may be unnecessary: it makes the output have units of 1/6 bits). Both of these `train` files were used for homology searches.

Finding matches to conserved deuterostome DNA

After comparing invertebrate DNA to the chimaera regions, there were 45 chunks of the chimaera regions conserved in at least one invertebrate. Most of these chunks are conserved in lancelet or acorn worm.

To boost sensitivity further, the searches were repeated against these chunks of chimaera, lancelet, and acorn worm. The chunks were first extended by 50 bases in both directions. Then, protein-coding regions and RNA genes of the three animals were extracted like this:

```
awk -F"\t" '$3 ~ /CDS|rRNA|tRNA|snRNA|snoRNA|guide_RNA/' genomic.gff
```

and cut from the conserved chunks. The chunks were indexed:

```
lastdb -c -uMAM8 -S2 ddb chimaera-lancelet-acornWorm-chunks.fa
```

Then similarities were found using `lastal` as above.

Thus, the searches were against deuterostome DNA (from chimaera, lancelet, and acorn worm). In future, more DNA conservation can probably be found by using non-deuterostomes too. The present study stopped here, to avoid risk of "transitive catastrophe": see for example stony coral and bootlace worm in the *Sp5* promoter alignment.

Multiple sequence alignment

In the end, after finding the conserved segments, the DNA sequences were aligned with the `linsi` method in MAFFT v7.520 [7]:

```
linsi sequences.fa > alignment.fa
```

Human genome-wide predictions of which enhancers regulate which genes

We used the file `AllPredictions.AvgHiC.ABC0.015.minus150.ForABCPaperV3.txt` from https://www.engreitzlab.org/resources/.

Transcription factor binding signals

The DNA sequences were scanned with transcription factor binding site profiles from the JASPAR database (non-redundant vertebrate profiles in JASPAR CORE 2022) [8]. The scanning was done by FIMO version 5.5.2 [9].

miRNA target signals

Potential miRNA binding sites were found with TargetScan Release 8.0 [10].

References

1. Frith, MC & Noé, L. Improved search heuristics find 20 000 new alignments between human and mouse genomes. *Nucleic Acids Res.* **42,** e59–e59 (2014).
2. Hamada, M, Ono, Y, Asai, K & Frith, MC. Training alignment parameters for arbitrary sequencers with LAST-TRAIN. *Bioinformatics* **33,** 926–928 (2017).
3. Frith, MC & Kawaguchi, R. Split-alignment of genomes finds orthologies more accurately. *Genome Biol.* **16,** 1–17 (2015).
4. Frith, MC. Paleozoic Protein Fossils Illuminate the Evolution of Vertebrate Genomes and Transposable Elements. *Mol Biol Evol.* **39,** msac068 (2022).
5. Frith, MC, Park, Y, Sheetlin, SL & Spouge, JL. The whole alignment and nothing but the alignment: the problem of spurious alignment flanks. *Nucleic Acids Res.* **36,** 5863–5871 (2008).
6. Frith, MC. A new repeat-masking method enables specific detection of homologous sequences. *Nucleic Acids Res.* **39,** e23–e23 (2011).
7. Katoh, K & Standley, DM. MAFFT multiple sequence alignment software version 7: improvements in performance and usability. *Mol Biol Evol.* **30,** 772–780 (2013).
8. Castro-Mondragon, JA *et al.* JASPAR 2022: the 9th release of the open-access database of transcription factor binding profiles. *Nucleic Acids Res.* **50,** D165–D173 (2022).
9. Grant, CE, Bailey, TL & Noble, WS. FIMO: scanning for occurrences of a given motif. *Bioinformatics* **27,** 1017–1018 (2011).
10. McGeary, SE *et al.* The biochemical basis of microRNA targeting efficacy. *Science* **366,** eaav1741 (2019).
11. Kumar, S *et al.* TimeTree 5: an expanded resource for species divergence times. *Mol Biol Evol.* **39,** msac174 (2022).
12. Engström, PG, Fredman, D & Lenhard, B. Ancora: a web resource for exploring highly conserved noncoding elements and their association with developmental regulatory genes. *Genome Biol.* **9,** 1–12 (2008).
13. Morgan, M, Iaconcig, A & Muro, AF. CPEB2, CPEB3 and CPEB4 are coordinately regulated by miRNAs recognizing conserved binding sites in paralog positions of their 3′-UTRs. *Nucleic Acids Res.* **38,** 7698–7710 (2010).
14. Vetere, G *et al.* Selective inhibition of miR-92 in hippocampal neurons alters contextual fear memory. *Hippocampus* **24,** 1458–1465 (2014).

15. Margvelani, G, Meparishvili, M, Kiguradze, T, McCabe, BJ & Solomonia, R. Micro-RNAs, their target proteins, predispositions and the memory of filial imprinting. *Sci Rep.* **8,** 17444 (2018).

16. Takada, H *et al.* The RNA-binding protein Mex3b has a fine-tuning system for mRNA regulation in early *Xenopus* development. *Development* **136,** 2413–2422 (2009).

17. Yang, L *et al.* The human RNA-binding protein and E3 ligase MEX-3C binds the MEX-3–recognition element (MRE) motif with high affinity. *J Biol Chem.* **292,** 16221–16234 (2017).

18. Umehara, T *et al.* Body temperature-dependent MicroRNA expression analysis in rats: *Rno-MiR-374-5p* regulates apoptosis in skeletal muscle cells via *Mex3B* under hypothermia. *Sci Rep.* **10,** 15432 (2020).

Tables

Table S1: Genomes with detected regulatory elements (size means number of a/c/g/t bases in the file)

Animal	Phylum	Scientific name	Genome	Size (bp)	Has gene annotation
human	chordate	*Homo sapiens*	`hg38_no_alt_analysis_set`	2934876451	yes
chimaera	chordate	*Callorhinchus milii*	`GCF_018977255.1`	991467956	yes
lancelet	chordate	*Branchiostoma floridae*	`GCF_000003815.2`	487128766	yes
acorn worm	hemichordate	*Saccoglossus kowalevskii*	`GCF_000003605.2`	642223636	yes
sea urchin	echinoderm	*Strongylocentrotus purpuratus*	`GCF_000002235.5`	921518293	yes
lobster	arthropod	*Homarus americanus*	`GCF_018991925.1`	2291242282	yes
centipede	arthropod	*Strigamia maritima*	`GCA_000239455.1`	173599458	
millipede	arthropod	*Glomeris maerens*	`GCA_023279145.1`	149766888	
scorpion	arthropod	*Centruroides sculpturatus*	`GCF_000671375.1`	863041242	yes
tick	arthropod	*Ixodes scapularis*	`GCF_016920785.2`	2226616818	yes
horseshoe crab	arthropod	*Limulus polyphemus*	`GCF_000517525.1`	1705786612	yes
sea spider	arthropod	*Nymphon striatum*	`GCA_016618385.1`	744500873	yes
velvet worm	onychophora	*Euperipatoides rowelli*	`GCA_003024985.2`	1744674727	
cactus worm	priapulid	*Priapulus caudatus*	`GCF_000485595.1`	436582939	yes
chiton	mollusc	*Acanthopleura granulata*	`GCA_016165875.1`	544712841	
sea hare	mollusc	*Aplysia californica*	`GCF_000002075.1`	737797481	yes
abalone	mollusc	*Haliotis rufescens*	`GCF_023055435.1`	1334438814	yes
owl limpet	mollusc	*Lottia gigantea*	`GCF_000327385.1`	298894987	yes
oyster	mollusc	*Crassostrea gigas*	`GCF_902806645.1`	647867327	yes
nautilus	mollusc	*Nautilus pompilius*	`GCA_018389105.1`	729019367	
octopus	mollusc	*Octopus bimaculoides*	`GCF_001194135.2`	1986279820	yes
solenogaster	mollusc	*Wirenia argentea*	`GCA_025802215.1`	528706996	
peanut worm	annelid	*Sipunculus nudus*	`GCA_026874595.1`	1426680931	
sandworm	annelid	*Alitta virens*	`GCA_932294295.1`	671131125	
Satsuma tubeworm	annelid	*Lamellibrachia satsuma*	`GCA_022478865.1`	664980950	yes
shingle tubeworm	annelid	*Owenia fusiformis*	`GCA_903813345.2`	499118861	yes
shamisen shell	brachiopod	*Lingula anatina*	`GCF_001039355.2`	389003419	yes
horseshoe worm	phoronid	*Phoronis ovalis*	`GCA_028565635.1`	325077420	
bootlace worm	nemertea	*Lineus longissimus*	`GCA_910592395.2`	391166316	
carnation coral	cnidaria	*Dendronephthya gigantea*	`GCF_004324835.1`	286150628	yes
stony coral	cnidaria	*Acropora millepora*	`GCF_013753865.1`	475342477	yes
lace coral	cnidaria	*Pocillopora damicornis*	`GCF_003704095.1`	225744106	yes
starlet sea anemone	cnidaria	*Nematostella vectensis*	`GCF_932526225.1`	269383238	yes
pale anemone	cnidaria	*Exaiptasia diaphana*	`GCF_001417965.1`	210157303	yes

Table S2: Genomes analyzed but no regulatory elements detected

Animal	Phylum	Scientific name	Genome	Size (bp)	Has gene annotation
fruit fly	arthropod	*Drosophila melanogaster*	`GCF_000001215.4`	142573024	yes
bee	arthropod	*Apis mellifera*	`GCF_003254395.2`	223937270	yes
bristletail	arthropod	*Machilis hrabei*	`GCA_003456935.1`	1320889507	
roundworm	nematode	*Caenorhabditis elegans*	`GCF_000002985.6`	100286401	yes
jawless leech	annelid	*Helobdella robusta*	`GCF_000326865.1`	215435648	yes
planarian	platyhelminth	*Schmidtea mediterranea*	`GCA_022537955.1`	773867583	
flatworm	platyhelminth	*Macrostomum lignano*	`GCA_002269645.1`	762829307	yes
brown bryozoan	bryozoa	*Bugula neritina*	`GCA_010799875.2`	214708255	yes
hydra	cnidaria	*Hydra vulgaris*	`GCF_022113875.1`	817163865	yes
trichoplax	placozoa	*Trichoplax adhaerens*	`GCF_000150275.1`	94748975	yes
sea walnut	ctenophore	*Mnemiopsis leidyi*	`GCA_000226015.1`	150338246	
comb jelly	ctenophore	*Bolinopsis microptera*	`GCA_026151205.1`	265424760	
sea gooseberry	ctenophore	*Hormiphora californensis*	`GCA_020137815.1`	110660632	
cigar comb jelly	ctenophore	*Beroe ovata*	`GCA_946803715.1`	84911038	
demosponge	porifera	*Amphimedon queenslandica*	`GCF_000090795.2`	143136305	yes
glass sponge	porifera	*Oopsacas minuta*	`GCA_024704765.1`	60959430	yes

Table S3: Other genomes used in this study

Animal	Phylum	Scientific name	Genome	Size (bp)	Has gene annotation
lamprey	chordate	*Petromyzon marinus*	`GCF_010993605.1`	1074036726	yes
hagfish	chordate	*Eptatretus burgeri*	`GCA_024346535.1`	1507324335	
Bahama lancelet	chordate	*Asymmetron lucayanum*	`GCA_001663935.1`	452764958	
Hawaiian acorn worm	hemichordate	*Ptychodera flava*	`GCA_001465055.1`	1126321642	
sea cucumber	echinoderm	*Apostichopus japonicus*	`GCA_002754855.1`	798993713	yes
robber fly	arthropod	*Machimus atricapillus*	`GCA_933228815.1`	268640068	
woodlouse	arthropod	*Armadillidium nasatum*	`GCA_009176605.1`	1223004717	yes
sea butterfly	mollusc	*Limacina bulimoides*	`GCA_009866985.1`	2900980226	
top snail	mollusc	*Phorcus lineatus*	`GCA_921293015.1`	957783961	
scallop	mollusc	*Mizuhopecten yessoensis*	`GCF_002113885.1`	907590157	yes
firefly squid	mollusc	*Watasenia scintillans*	`GCA_015471945.1`	649114004	
medicinal leech	annelid	*Hirudo medicinalis*	`GCA_011800805.1`	156118814	
waratah anemone	cnidaria	*Actinia tenebrosa*	`GCF_009602425.1`	214400470	yes
zoanthid	cnidaria	*Epizoanthus planus*	`GCA_025388665.1`	215910112	

Table S4: Invertebrate genomes that were compared to each other

Given to `lastdb`	Given to `lastal`
stony coral	waratah anemone
sea hare	sea butterfly
lancelet	Bahama lancelet
lancelet	acorn worm
oyster	scallop
fruit fly	robber fly
millipede	centipede
abalone	top snail
jawless leech	medicinal leech
lobster	woodlouse
Satsuma tubeworm	shingle tubeworm
horseshoe crab	scorpion
shamisen shell	horseshoe worm
sea walnut	sea gooseberry
starlet sea anemone	zoanthid
octopus	firefly squid
acorn worm	Hawaiian acorn worm
sea urchin	sea cucumber

Evolutionary tree

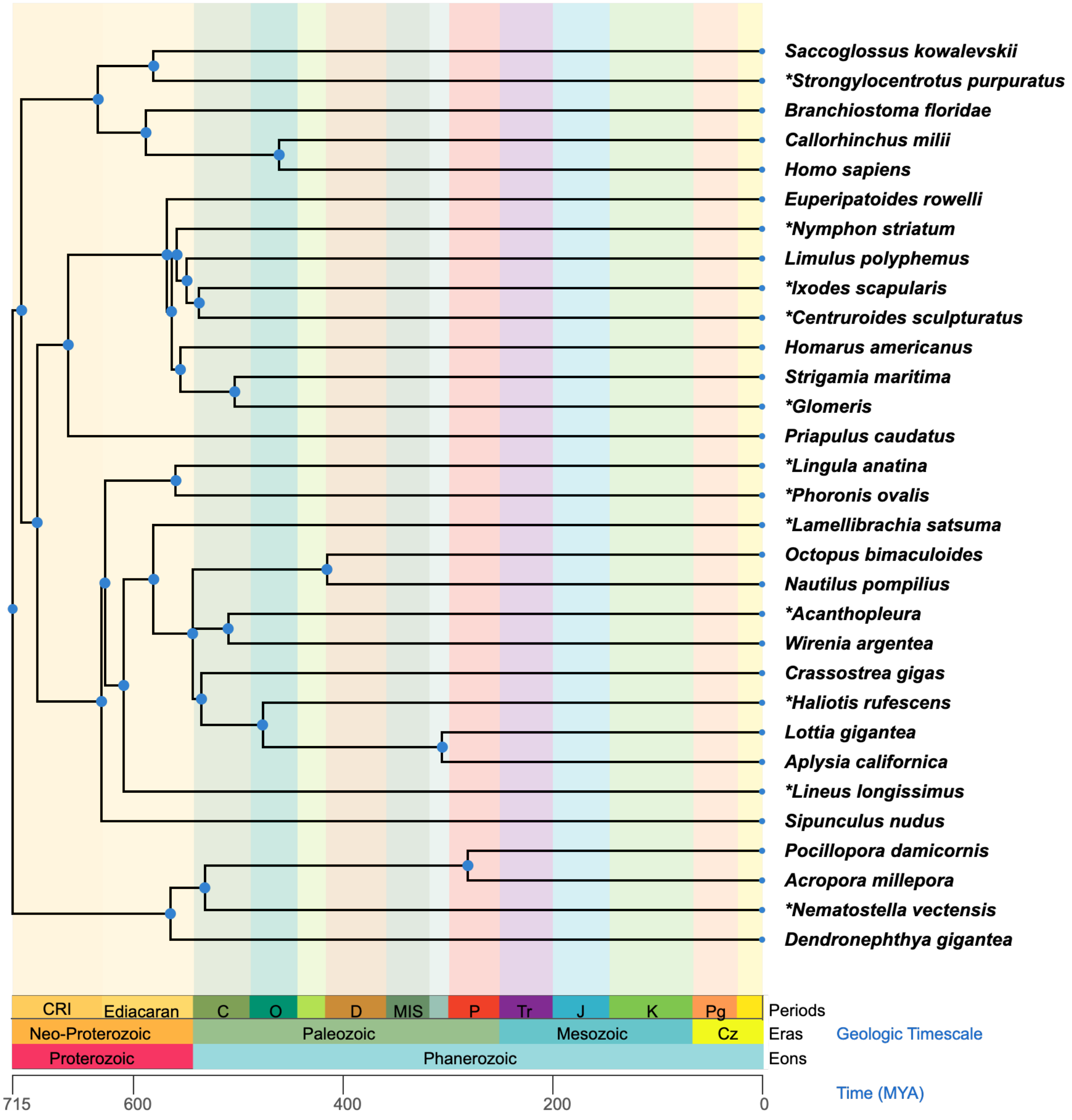

Figure S1: Evolutionary tree of the animals with detected regulatory elements, according to TimeTree [11]. ⋆ means that TimeTree used a closely-related taxon. Three animals are missing (not found by TimeTree): *Alitta*, *Owenia*, and *Exaiptasia*.

Non–protein-coding DNA segments conserved in vertebrates

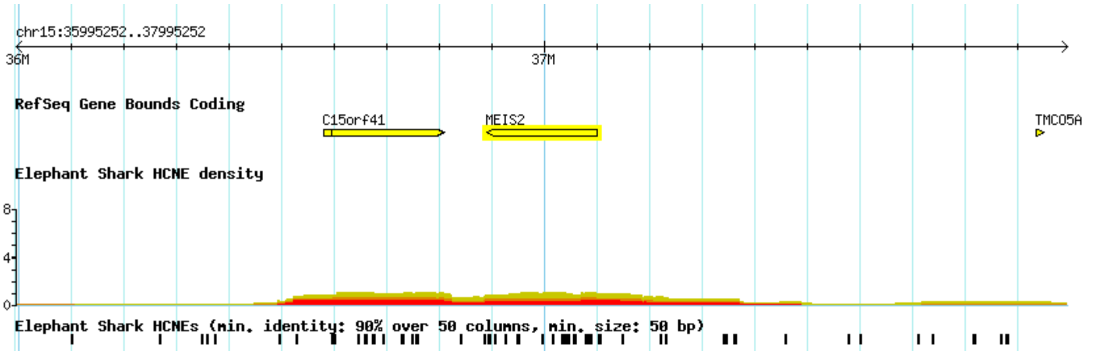

Figure S2: Highly conserved noncoding elements (HCNEs) around the human *MEIS2* gene. These elements are conserved between human and elephant shark (chimaera, *Callorhinchus milii*), according to Ancora [12].

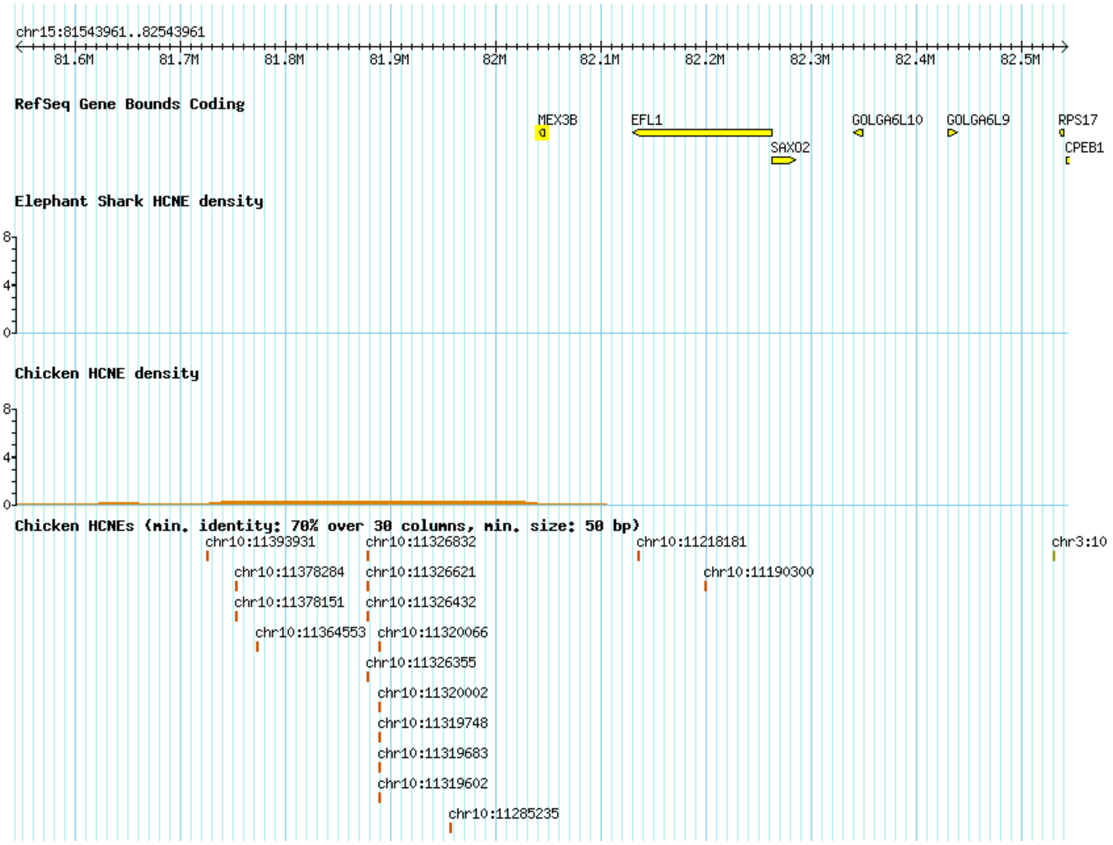

Figure S3: Ancora HCNEs around the human *MEX3B* gene.

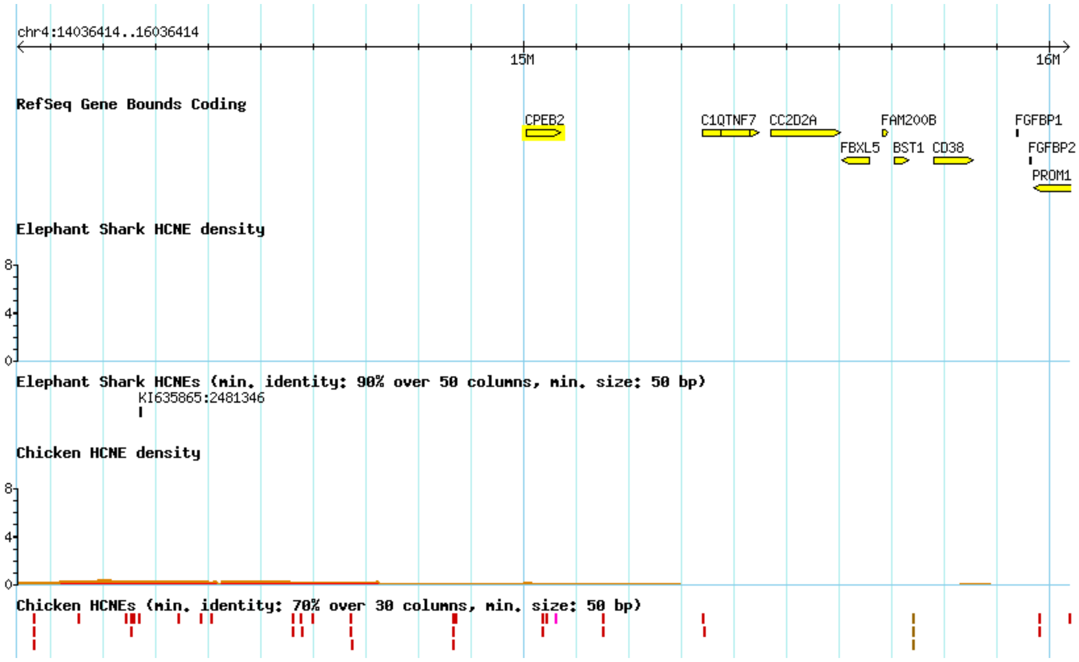

Figure S4: Ancora HCNEs around the human *CPEB2* gene.

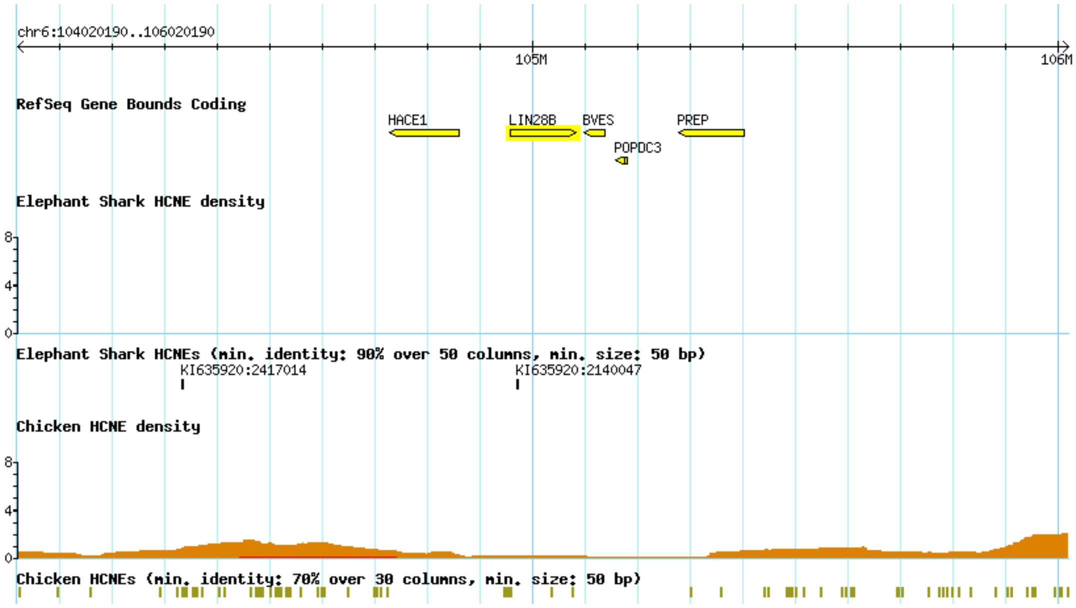

Figure S5: Ancora HCNEs around the human *LIN28B* gene.

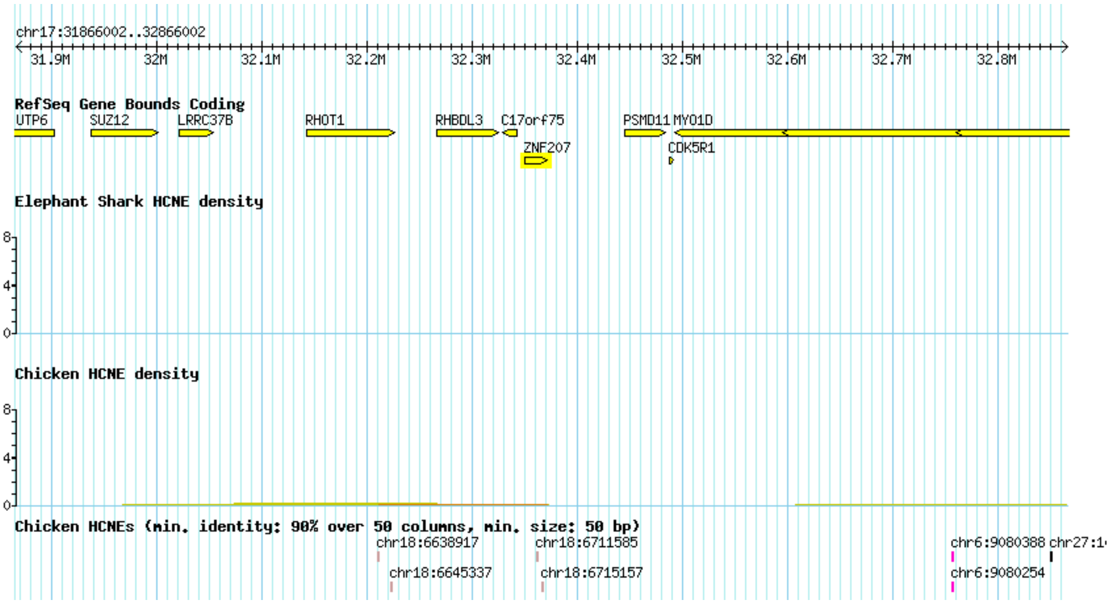

Figure S6: Ancora HCNEs around the human *ZNF207* gene.

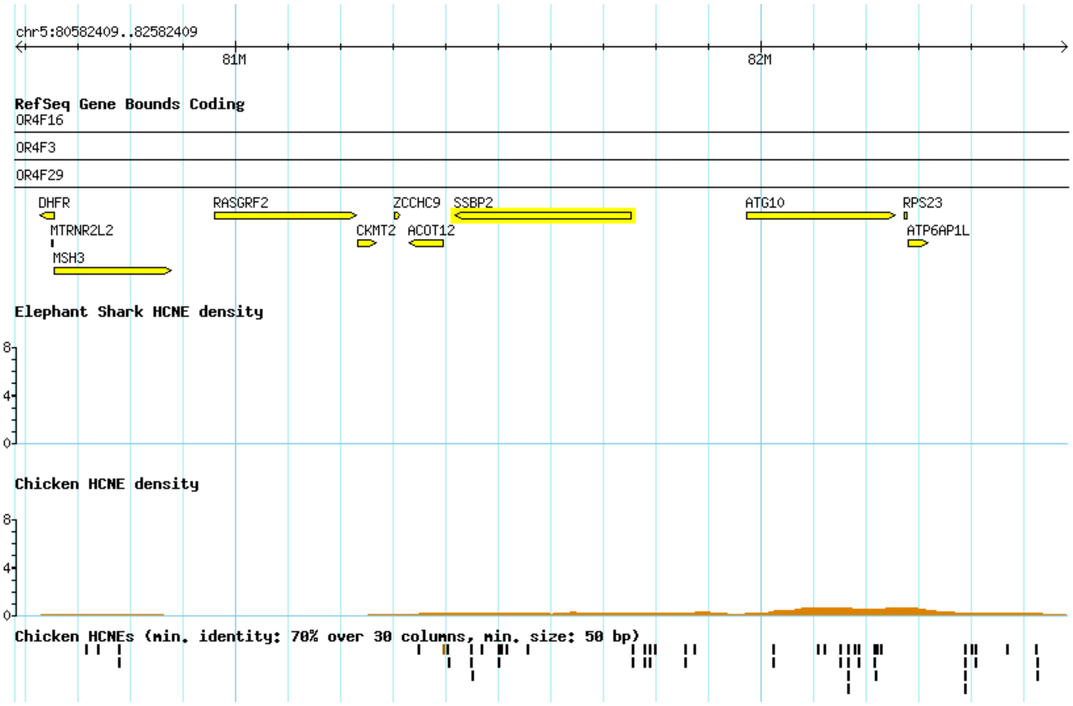

Figure S7: Ancora HCNEs around the human *SSPB2* gene.

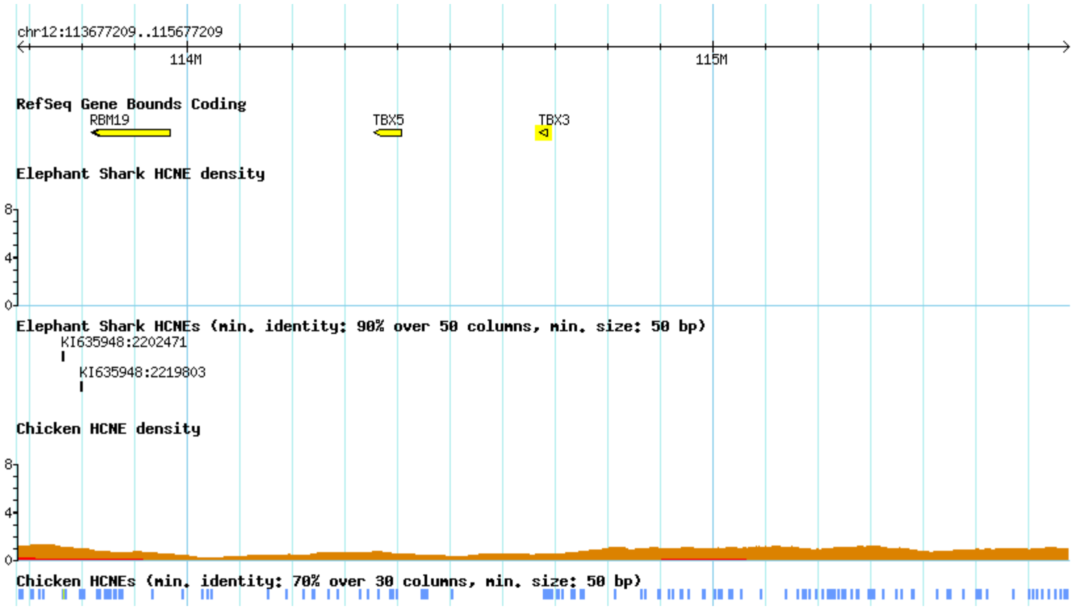

Figure S8: Ancora HCNEs around the human *TBX3* gene.

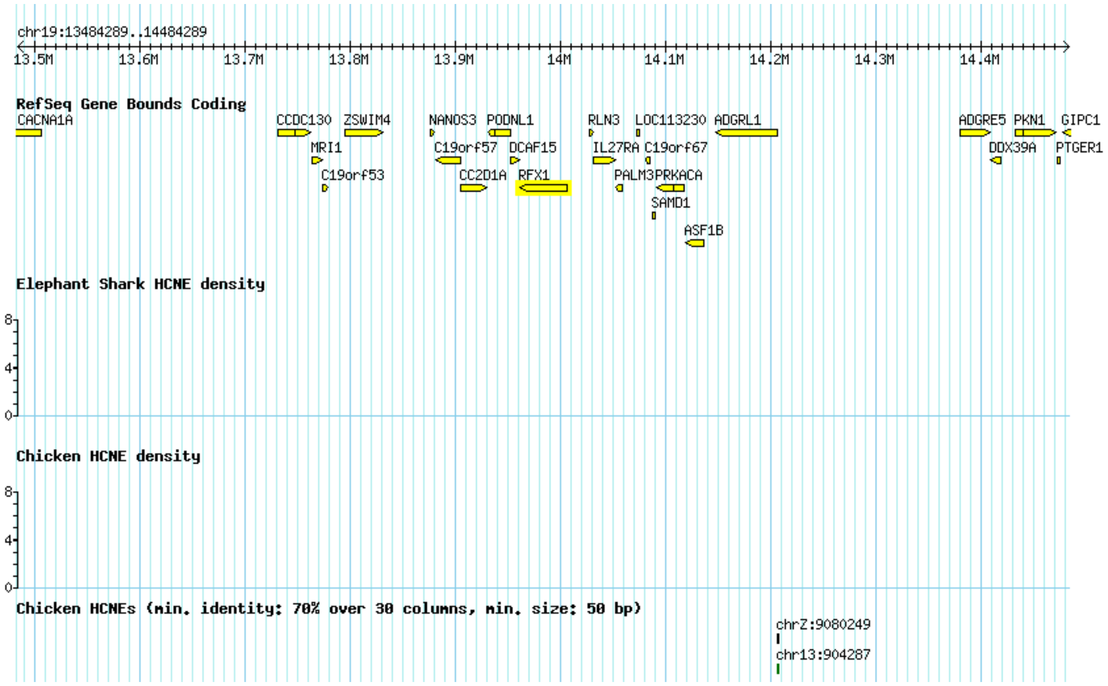

Figure S9: Lack of Ancora HCNEs around the human *RFX1* gene.

Alignments

Above each alignment are shown transcription factor binding motifs that are conserved in most of the sequences. Some are labeled by name of the (family of) transcription factor. Others are labeled by “box” or “element”:

- E-box: `canntg`, bound by many bHLH (basic helix-loop-helix) transcription factors and some zinc finger transcription factors.
- CCAAT: CCAAT-box, bound by NF-Y.
- WRE: Wnt response element, bound by TCF/LEF proteins, the consensus is said to be `ctttg` or `cctttgww`.
- NRRE: nuclear receptor response element, the consensus is said to be `aggtca` or `(a/g)g(g/t)tca`.
- GC-box: bound by Sp transcription factors, the consensus is `gggcggg` or similar.
- CArG-box: `ccwwwwwwgg`, bound by transcription factor Srf.

```
                                                                                                                                                   Sox___
human GSX1       --gggtctgtta-------gacggtgcggtgcctgcagctgatttgatgggttggaaatgcgcacgaaacaaaatacttgaat-cctgaaaaagaccctttgtaagtttctt-tagaaatcaagcaattctgcatgacaa
human GSX2       --gggtctggca----ggcgcagaggtgccgtatgcaaatggttc------actattaggcgagaaaga-----------aatacttcaaatggccctttgtaaccttctat-agaaaatcgaggtactctgcatgacaa
chimaera gsx1    -----tctggta-------gacggtgcgatgcacgcagctgatttgatgagttggaaatacgcacgaa--aaaatacttgaat-cctgaaaaagatcctttgtaagtttctt-tagaaatcaagcaattctgcatgacaa
chimaera gsx2    --gggtctggta-------ggcgaggtgggatatgcaaatga-----------caggatattcgcaaggaaaaatactcgaatcctccaaatggctcttttgtgagtttctt-tagaaatcaagcaattctgcatgacaa
lancelet         cccaacctgctgtcggggggcagagataattttccaaaatggagt-----ggaaaatagacc--------aaaatatatagca-gcgcgccgggctttgtgctgcttgtatataagaaaacaacatattctacatgacaa
acorn worm       cgcaatctgctg--gccgcagccagataatttatgcaaatga-----------ggcgcggcaacgccag-aaaatacttgaaaccccaaaaagcctctttgtttcttctgta-aagaaaaccaacaattgtgcataacaa
velvet worm      -gcaatatgcta-gacggaggggggataatttatgcaaatga------------agtaggcgctctgga-aaaatactttaaa-cctgaaaagcctctttgtgccttatgta-aagaaaaccaacaattgtgaataacaa
cactus worm      ----------tg--gctagagcccgataatgtatgcaaaata---------ttcgagacactctctaga-aaaatagtttaat-cgtgaaaagctcttttgtgcaaagcgtc-aaagcagccctcagtgttgaacaacaa
chiton           -------------------------ataatgtatgcgaatgtt--------ctcggcacacttatagca-ctgatacatggatccccaaaaggtctttttctccgttccata-tagaaagttaagaaacgtcgacaacaa
abalone          -----------------------aggcaatgtatgcaaatga-------------gtgtgcacccttagaacactgcccaaga-tttccacagattctttgt-atttctagc-aaagaaaccaacaatttgcagcgacaa
nautilus         --------------------------------atgcaaatga-----------gaaaaaacgcgcttcg-caactactctaaa-gctgaaaagattctttgtacggcctgca-aagaaaaccaacagtcgtgaataacaa
octopus          ------------------------------tcatgcaaatga-----------gaaaatgctggcttga-aaactacttttgg-cctgaaaagcttcattgtacacggtgta-aagaaaaccaacaatcgaaaataacaa
peanut worm      ------------------------------------------------------------------------------------------agaaagcctttgtgcctcccca-aagaaactcaataaatgcgaataacaa
shamisen shell   -----------------------------------------------------------------------------------------aaagaaccttttgtatgttagta-aggaacaccagcctcgttgaatgacaa
horseshoe worm   ----------------------------------------------------------------------------------------aaagaagtctttgtgtcctatgta-aagaaaaccaacaattgaaaataacaa
bootlace worm    --caacctgctc--tctctgcccagataatttatgtaaatga------------acggggcgcgctgga-aatatactttaaa-catgaaaagctgcattgtgttttgtgta-aagaaaaccaacaattgtgaataacaa

                     Sox_____
                 Hmx________________
                 ------                                   E-box_
human GSX1       ag--aacaattaat-aagccgtcttttcaca----aaccagcagctggtttccccgtcaaggaaagttggaaaaaattcaggctgaatgcgcgcagaagctcttt------------
human GSX2       ag--cacaatt----aagctttctattcagg---agttctgcagctggtagcccat--tgggaaagtgggaggaaa-----------------------------------------
chimaera gsx1    ag--aacaattaat-aagccgtcttttcaca----aaccagcagctggtttgccgt--caggaaagttggaaaaaattcaggctgaatgcgcgcaaagctcttt-------------
chimaera gsx2    ag--aacaattaat-aagccgtcttttcacg----agctagcagctggtaacccgt--ggggaaagtaggggaaaatcagagatgaatgcgcgcaaaagtt----------------
lancelet         aaggggcgattgat-tacttggtgggtttcc------acggcagatggttaagg-----gggagaggtggcgggaggg---------------------------------------
acorn worm       ag--aacaattaat-aagttgttttttcacc----aaacggcagatggttcgaacg--agaagaagggagcgaaaagttgaaatgcctcgctggaaacaaaatgcgcgctttttaca
velvet worm      ag--aacaattaat-aagttgtttttctacc----aaacggcagatggttcgagcg--actgtaacatactcaaaagttgatatgcttttgcgaaactttt-tgcgctccttttaca
cactus worm      ag--aacaattcac-aagttgtttttccacc----aaacgccagctggttaggccg--ctcgtaacatattcaaacgtcgagaggcgctgacgtaaccgcgatgc------------
chiton           ag--actaattaataaaattgtttttctacc----aaccggcagatggtttggacg--acggga-----------------------------------------------------
abalone          ag-aaacaattgcc-atgttgtt----------------------------------------------------------------------------------------------
nautilus         ag--aacaattaat-aagttgtttttctacc----aaacggcagatggctgg-----------------------------------------------------------------
octopus          ag--aacaattaat-aagttgtttttctacc----aaacggcagatggctgg-----------------------------------------------------------------
peanut worm      ag--acctgttaat-aagttggtttttgccttcaatacggcagatgttt-------------------------------------------------------------------
shamisen shell   ag--aacaattaat-aacctttttttccgca----aaacggcagatgttttctcg--------------------------------------------------------------
horseshoe worm   ag--aacaattaatcagaaagttctttcgtc----atacggcagatggt--------------------------------------------------------------------
bootlace worm    ag--aacaattaat-accttgcttttctggc----aaacggcagatgtccaacccg--atgggaacatatttaaaacttg-------------------------------------
```

Figure S10: *Gsx* enhancer

```
                                                                                                                                                 CCAAT
human HMX3          ------------------------------------------------------------------------------------------ttcgcctgcctaatcccatttgccattgtacgcgcccaatcgccgt-ata
chimaera hmx1       ------------------------------------------------------------------------------------------ttttcttgagtaatcctatttgccattgtgcccgtccaatcgtgga-ata
chimaera hmx3       -------------------------------------------------------------------------------------ctctccccccttcagtaatcccatttgccattgtgcgtacccaatcgccgt-ata
lancelet            aaccggttgttgggatcgcccaataagaggggttttccaccactcgtccccattgttcaagatttgaga-ggtgtggtcacttgagcttttcgttcttcgtaatccaattacccattgtgtgtgtccaatcaccga-gtc
acorn worm          ---------------tcgcccaataagggtatgttttcaccactctgt-gcattgttatccatataagaaggcgtggttacagtggagtcgctttcactgtaatcctattgcgtcgtccccgtggccaatcacaga-gct
abalone             -------------------------------------------------cgatcgtcgatgattggagaggcgtggtgtactggaggattcctctttcattaatccgattagcttttgtgtttggccaatcagtgatttt
shamisen shell      --------------------------------------------------------------------------------------------------tctaatcctattatgtattgtcctcgaccaatcgc-ga-atg
stony coral         ---------------------------------------------------------------------------------------------tttctcatagttccattagccgtcattttagaccaatcacagc-agc
lace coral          --------------------------------------------------------------------------------tgattattcgagctttctcgtggttccattagtcgtcaccctggaccaatcacgga-cgc
starlet sea anemone -------------------ccaatcacagtggg-ttttatcagttt---tcccggttgtttgcaaagaagggggcggggaagtgcattttagctttctcacggtcccattagatgtattgctcgtccaatcagcgc-tct
pale anemone        ----------------------------------------------------------------------------------tgcattttctctttctcacggttccattagacacaaaggtcgaccaatcatagt-aca

                                                                 EVX_______
                                  BARHL_____     NR4A______     POU6______
human HMX3          ctcccc---------gcatttaacttggatgacattttgatt--tcatcattagcatc---------------cggcgccggattgacgc---
chimaera hmx1       ctctttgcttgttaagcatttaacttgcatgacatttttatt--tcatcattatcatttttcccccttct-----------------------
chimaera hmx3       ctttct---------gcattgaacttgtgtgacatttttatt--tcatcgttatcacc---------------cggcgccggattgacgc---
lancelet            gttttcagttgtcaagcatttaacttgtatgacacgtttatt--tagacgttattattgttatctctgctggacggcgccatttcgactcagc
acorn worm          tcattagcttgtcacccatttaacttgcacgccacttgtatt--tatatgttattagcgtg---ttagatgagcggcgccaatacgactc---
abalone             gcttgaagttgtcactcgtttaa----------------------------------------------------------------------
shamisen shell      cagtccatttgtcatccatttaacttctctgctactcttttctcttgtagttattatcgct---ccctgtgatcggcgccatcacggttaagc
stony coral         gcatttgcatgtcagacgtttaacaagtttgacatttttattcgtgatcattatcaccggc------ttaaattgtcgcctcgcatgc-----
lace coral          gcatttacatgtcagacgtttaacaagtttgacatttttattcttgatcattatcact-----------------------------------
starlet sea anemone ccgtttgcttgtcaaccgtttaacaagtttgacgtttttattcgcgatcattatcacaggc------ttaaaacgtcgccacttaagctca--
pale anemone        gttcttccttgtcaaacgcttaacaagtttgacatttttattcgcgatcattatcatcgac------ttaaaacgtcgccactt---------
```

Figure S11: *Hmx* enhancer

```
                                                                MEIS___   NFI______________                                        FOXO___
human MEIS1     -cagagcggatgatcattcattcaccacgttgacaacctcgcctgtgattgacagctggagtggcagaaagccatgagatttg-----gtagttgg---gtctgaggggcgc---tcttttttttccttt---tctttct
human MEIS2     tcatcatcaaccatcattcattcactaccttgacattccgggctttgattgacagctggagtggcaaaaagccatgaaacacg-----acagttcg---gttacatgtgggc---tgctgacgggccgct---cgtaacc
chimaera meis2  --acggcagaacatcattcattcaataccttgacaaccc-tgctctgattgacagctggagtggcaaaaagccatgagagacg-----acagtttt---gttacaagcgggc---tgttgacgggccgat---cgtaacc
lancelet        tcatgacagatcatcagtcattcagtactatgacaaccg-cgctttgattgacagcttgagtggcaaaaagccacgagacacg-----acagtttt---gttaca-ggcgct---tgttgacgggccgacattcatttcc
acorn worm      ---tgacatctcatcactcacgtccttccatgacaacct-cactttgattgacagcttgaatggcaaaaagccacgagacacg-----acagtttt---gttacagggctgt---tgttgatgggccgacataggtttcc
sea hare        ---tgacagctcatttattatccgggccctcgcggacggctcgtttgattgacagatttagtatca-gcatccacgagtcgcg-----acaggccctgttgttcgagtgtgctcgtgttgacgggc-------------
abalone         ---tgacagctcatttgttatcccgttctatagtaaccc-tggcttgattgacagatttagtggta--------------------------------------------------------------------------
oyster          ---tgacagctcgtacattatttcttgctagagtaacgt-ggttttgattgacatatt--gcgagaaatagccatgagttaag-----acaggtt---------------------------------------------
shamisen shell  --atgacagctcatttgttactcgtcactatggcaacgg--cggatgattgacagcctcgctgg-aagaggccacgagcatcg-----acagttta---gtt--------------------------------------
carnation coral ------------------------cctctatggaaactt-tagtttgattgacaggagatatggcaggcagccatgatacaaaccttgacaattc----gttacgact-----tgtgatgacgggggaatatgctatggg
stony coral     ---------------------------------------------tgattgacagacttgt-ggccaaaagccacgagacagg-cttgacaattc----gttactagt-----tgtgttgacggccgtacacatcttttc
lace coral      ---------------------------------------------tgattgacagacttgt-ggccaaaagccacgagacagg-cttgacaattc----gttactagt-----tgtgttgacggccgtacacatcttttc
pale anemone    ---------------------------------------------tgattgacagatcagtggggcaagagccacgagacaag-cttgacaatcc----gttacgggt-----tgtgttgacgggaatacacatctttct

human MEIS1     ttc------------------tttcttttttttttttaaactgattttt--------------------
human MEIS2     ttcagttcgggggcttgacaattttttttcttctttttcttctttttcttttcttt--------------
chimaera meis2  ttcagt-------------aatttttttttttttttttggcttgacatttttttt--------------
lancelet        ttctgt-------------tgcctgacattcttgtgtaagcttagaaagtccccggtacgttcaaactg
acorn worm      ttctgt-------------tagctgacacatttctgtaagcttagaaagtccccggcctgttcaaattg
sea hare        ---------------------------------------------------------------------
abalone         ---------------------------------------------------------------------
oyster          ---------------------------------------------------------------------
shamisen shell  ---------------------------------------------------------------------
carnation coral ttctcatt-----------tggttgacgggt---tgtaagcttagagagcc------------------
stony coral     cttacatt-----------gagttgacgggt---tgtaagtttagaaagctatccaactgt--------
lace coral      cttacata-----------cagttgacgggt---tgtaagtttagaaagctatccaactgt--------
pale anemone    tctgca--------------acttgacgtgt---tgtaagtttagaaag--------------------
```

Figure S12: *Meis* promoter

```
                                    WRE__                       E2F_________                Smad_____
human MSX1       ----------------cccgtgcgccgggtgcagcctttgat-ccggccg-----------ccgctggcgccttaaaa--caacat-cctagtctgcctatt-----aggcgcgctgaggaatcgtgacacattgtttat
chimaera msx1    ----------------ctctccctgaagtcgcctcctttgat-cgcgccg-----------ctgctggcgcctaaaaa--caacataacttgtctgcctatt-----aaaggcactgactaatcgtgacagattgcttat
chimaera msx2    ----------------ccccgggggcgggggggggctttgat-caggccg-----------atcctggcgcctatgaa--caaccccacttgtctgcctctt-----aacttgtctgactaatggtgacagactgtttat
chimaera msx3    --------------tctccctgaggtagcgtctcggtttgat-cccaccg-----------aagctggcgccttccaa---aacataacttgtctgcctatt-----aaatgccttgacttatcttgaagaattgc----
lancelet         gggtaagtgttacatattcggagtgattgtggtccctttgat-cccgccg-----------ttcgtggcgcctagaaa--caacacaacttgtctgcgcatt-----gaaatatctgacaaatcaggtcagattgtttat
acorn worm       gggtaactgttccatatgctaagcaatgcatttctctttgat-cgggccgttaacgctctcttagtggcgcctgtacactcactaaaggatgtctgcttttttttaaaaaaaggatggcaagtcgtgatggattatttgt
sea urchin       --------------------------------------------------------------ttgtggcgccgactgc--cgtcctaagatgtctactcagt-----gaataaggtgacaaatggtgacggagtgtttgt
Satsuma tubeworm ----------------------------tggtctcctttgaa-gcgtccg-----------acgttggcgcctagcgc--gaacataacttgtctgtctatt-----cttcggcctgacaaatcgtgacagattgtttag
shamisen shell   ----------------ctatgcgcggggctgttttctttgatgtaccccg-----------accctggcgccttcttg--aaacttaacttgtctccctata-----gaaaagagtgacaagacttgactgcctgcttgc

                     MSX_____
human MSX1       ccccgcaattagcgatg------------------------------------------------------------------------
chimaera msx1    agtcgcaattagcaatgcagacgctc-ggcgg---------------------------------------------------------
chimaera msx2    ccgcgcggttacccagcaggtgccgc-gggg----------------------------------------------------------
chimaera msx3    -----------------------------------------------------------------------------------------
lancelet         cctcacaattagtactggctaggtca-caaggtaattgatcactc--------------------------------------------
acorn worm       cgagccaattagctctggcttggtcc-gagggtaattgatcacct----aaaggggtgtgtactcattgcaaataactcctttatcttc
sea urchin       acttgcaattagcctggctatgcctcctgaggtaattgatcgcccgaatagaaagagccgttcccactgaaaaaaccctctccctcctc
Satsuma tubeworm acgcacaattagcgacgccctgacgc-gg------------------------------------------------------------
shamisen shell   tcaagcaattag-----------------------------------------------------------------------------
```

Figure S13: *Msx* enhancer

```
                                                                                               E-box_ FOX_______                                   TEAD____
human SIX1     gtgcgcacttgctccagactttagggtattaactgatttttagagccgggtttaagcctccgttc---cggtcctcacacagctgccatgtcaata--agga-ctgtttcaagca---gaggcattccttctgggagggc
chimaera six1  gtgcgcgttcgtttggtgatttacggtacaatctgactccgatag--agggttaaggatcagttc---cggtccccaaacagctgccttgtcaata--aggatctgccacaagtg---gagacatccctgctgtcaggac
lancelet       -------------------taccttatacttactaatactgagtc--cgatccacgtattggttc---cggtatcgaaacagctgttttgtcaata--agaa-ctgtcaaacgaa---aaagcattccaacgatgccggt
velvet worm    ----------------------------------------------------------------------------------------------------------------ggg---gaagcattccgctaataccagt
shamisen shell -------------------taccttacactgatcaatttagagcaaaacgttagagcattggtccaagtagcactggaacagctgttttgtcaatattagcg-cagtctggggagcataaagcataccactaataccggc

                           MEIS___
human SIX1     --------------------------------------
chimaera six1  --------------------------------------
lancelet       gatgtactctacctgtcaaacattatctctaataacat
velvet worm    gatgtact-tacctgtcaaatattatctttacaaacat
shamisen shell -----------cctgtcaaatattatct----------
```

Figure S14: *Six1* enhancer

```
                                                     PKNOX__________
                                                  GFI1________
                                          Six________            RFX_____________                                                         CCAAT
human SIX2        --------------cccgccgaggctcgggttaccagtgactgacagcgtctccatggcgaataatttgactccgactattgtctggcgctggcaggcccc---gggtcagataaccggaccaatcagggcgcgggccgc
chimaera six2     --------------------------tcgggttactctgattgacagcgtctccatagcaaataatttgact-taactattgtcttatcctcaccatccgt----aggtcagatctccgaccaatcgtatgctacacaat
lancelet          atactcttttgacacctaagtgcatctcatgttacattgattgacattgtcgccatagtgaataatttagt--aaaccattggaataatatcgatatatcc--ggcgcgcacactcctgaccaatcacggcaaagcagct
acorn worm        acgttctgttgacacctcatcggcgatcaagtttcactgattgacgtggttgccatggcaaataattcgac--aaactattgtatgaatatctatatatccgtgg---cgagcaagcggaccaatcagggcagagtatct
velvet worm       ----------gacacctaaagcagggtagggtttcattgattgacggcgttgtcatgacaactaattttgt--caagccttctatgaatattgatatattggtggggtcggaagaaccaaccaatcagaagcgtgaataa
abalone           -------gtccacgcctcttgatgggtcgggtttca-tgattgacattgttgctatggcgatggattttca--caggcattgtgtgaataacgatatatccatggggtcgcgcgccagagccaatcagcggccagcagag
owl limpet        ------------cgcctcctgccctatcaggtttcagtgattgacgtcgttgctatagtaatagattttca--caagcattgtgtgaataacgatatatccacaggg---------------------------------
oyster            ------------------------------------ttgattgacgtcgttgctgtggttactaatttctc--acggcactgagagaataagcgatatcttctggggtcgccagaaacagccaatcatctgaaagtagtg
peanut worm       ------------cacctctcagcgtatcaggttacagtgattgacgtggttgccatggtgactaatttctt--gcagcattgtatgaatatcgatatagccggcgggtcgctgcgcacggccaatcacggcccggatgcc
sandworm          ------------------------tgtcgggtttcagtgattgacggcgtttccatggagaataatttggt--agagtattgcatgaatatcgatatgtccacggggtcgaggccatgggccaatcagcgtgcagccggc
Satsuma tubeworm  -------------------------atcgggtttccgcgatagacggtgttgccatggcaagtaatttgtc---gcgcgttgcatgaatatcgatatgtc----------------------------------------
shamisen shell    ------tatcaacaccccttcccgtatcaggtttcactgattgacgtggttgccatggtgtaccaactcac--taagtattgtatgaatatcgatattagcaaggggtcgggaagctgaaccaatcagcttctagcttac

human SIX2        cgcgcctcatgcccgcttag-aataatattattaaga-----------------------------------------------------------------------
chimaera six2     gactattcgtgccaacttgg-aataatattattggaaaaaaaagaaaccccccca------------------------------------------------------
lancelet          ggctattaatgtcgccctaa-aataataatgtcaaaaagtag--caaccccacacgttggcctgtttgtagtgtcgttttttccctgctacacacacacacacgcacg
acorn worm        ggctattaatgtcgcgttaa-aataataatgtcagaacgcgg--caaccccacacgttgggttatttgcactgttgcttctca-------------------------
velvet worm       aactattactgtcgctttaa-aataataatgtcaaagcatcc--cagccccacacgttggcttgttttcaagcttatctttcaccccgaagacgaatattcaagcagg
abalone           ggcca-------------------------------------------------------------------------------------------------------
owl limpet        ------------------------------------------------------------------------------------------------------------
oyster            aaccattactgcacctcta-----------------------------------------------------------------------------------------
peanut worm       gcctattactatcgccttga-aataataacgtcaagatgttg--gtctctcacatgttaactctgttt----------------------------------------
sandworm          ctcctgtaacgttgctttgaaaataataaggtagaggcagg----agcccc---------------------------------------------------------
Satsuma tubeworm  ------------------------------------------------------------------------------------------------------------
shamisen shell    ttccactaatgtcgccttaa-aataataacgccaagatgtg----agccccacacgttgggttgttttcac-------------------------------------
```

Figure S15: *Six2* promoter

```
                                                                         Nrf1___________          Yy1______                     HOX_______
human ZFHX3       tattgatccagaaggtggctaattagcccttt--cccgtcgtgcctgggtaatg-aagcacatgcgcactgaattaatcacagccatttttt------------gcaggaaactaattagcagaataaagatccaaagact
human ZFHX4       ------------------------------------------------------aaagacatgcgcactcaacttaat-cagccatttttttaaacagggctaaaacgataataattagcagaataaagacatatcggat
chimaera zfhx3    tattgatccgagggttcgctaattagccctttacccgtcgtgccttcgtaatg-aagcacatgcgcactgaattaatcacagccattttttg------------cacggaaactaattagcagaataaagatccatcgact
chimaera zfhx4    ---------------tcactaattacacctt--cccgtcgtgcatcggtaatgaagacacatgcgcactcaacttaat-cagccattttttttttacaggggc-------aaaataattagcagaataaagatatatcggat
lancelet          -----------------------tagcctat--cccgtcatgccc--atcgcg-acagacatgcgcgctgcgctaat--cagccatttttac------------acggaaaataattagcagaataaa---atatcgaat
acorn worm        -------------agatgtaaattgtctttc-gctta-----------------ttacgcatgcccattga-tgcctcccagccatttt-------------------ggaataattagcaaaataata--atatcgaat
sea urchin        -------------agattgggattaccttgc-accta-----------------ttacgcatgctcattgattgcgctcctgccattttac--------------acaaaaataattagcaaaacaat------------
abalone           ----------------------------------------------------------------------------------------------------------------taattagcgcaataaa---atatccact

human ZFHX3       ttttt-------------------------------------------
human ZFHX4       tttcatttc--ctttcctccttttccc---------------------
chimaera zfhx3    tttttcatt--gcgagtttcgccctcttttccgcc-----gcgatcgc
chimaera zfhx4    tttcatttc--cagtttccccttttccgttt---------gcggtcgc
lancelet          tttcatttg--gttatttctagtttctttctcggcgccgagcgcttct
acorn worm        tttcatttg--gttatttttagctctttatttttcacctagcatttgt
sea urchin        ------------------------------------------------
abalone           tttcatttactgttatttttagctgccgttttg---------------
```

Figure S16: *Zfhx3/4* promoter

```
                                                               NRRE__        NRRE__                   POU4__________
human EBF1        ----------------------ttttacttcctgtttcaa-----aggtcaaggcaggctgacctcaacagcagtgtggaggctgctgaattattcatatcattgactttgtgtca-----gcttccacatttgtggaga
human EBF2        ttctgtggtcactattattttcttggcctacccatcacaaaacagaggtcacagcaccatgaccttttcctggtggaatggactgcttaagaagtcacatggccaagtttctatcg----ggggtccacatttgtttcct
human EBF3        ---------------tgtttcctttcacttcctgtcacaa-----aggtcaaagagagccgacctttgc-tggcagtcctgctcactgaattattcatgggattgactttttgtca-----gcacacacagttgtgctct
chimaera ebf1     -----------------ttcccctccacttcctgtcataa-----aggtcgaggcagggtgacccctgcatcagcagcaaggttgctgaattattcatgcaattgactttgtgtca-----gcgtacacatttgtgcaaa
chimaera ebf2     ------------------------------------------------------------------------------------------------------------------------------cacatttgttcttg
chimaera ebf3     tttcttagtcaacattgcttcctttcacttcctgtcacaa-----aggtcaaagaaagctgacctttgc-tggcagcttcagttgttgaataattcatgtgattgactttttgtca-----gtgcacacagttgtgctct
lancelet          tg-------------tctcctaactcatttcttacttgcg-----gggtcaggctctactgaccttt---agcggcaccagtaccatgaattatttataaggcaaaattattagaa-----atgtacacagttgtgtttg
velvet worm       ------------------------------------------------------------------------------------cattaattattaatgaagaagcgttatgattagtgtggtacacacaactgcggatt
chiton            ---------------------------------------------------------------------------------------------------------------------------gtacacaggtgtcctct

                       ZNF24________                        E-box_
human EBF1        gggatgttttattcatttatctcatttagggttcttctctgcacctgtttctaccattaagtaccctgtaatttgcatttgatgctaatttagtt-tcatatcagcctggtccccccttgttaa-tttaatttt------
human EBF2        ccactcttttaatcagaatgctcatttaattct-attccagcacctgttc--agcaattaacaccctacgatttgcatcttaccctga-----------catttgctatctttccaa---ttagtttttatatcagtata
human EBF3        gggacattttattcatttacctcatttaaagcgcctttctgcacctgttc--aagtattaatatcgtgtaatttgggcctaatgccgattttgct-gaacactcattatatttttta---ttag-ctatatttccaaacg
chimaera ebf1     gggatgttttattcatttatctcatttaagggcctttcctgcacctgttcttaccactaagtacccagtgatttgcatttgatgctaatttagttatcgtatcggactggttttcctc--ttaa-tttcatttt------
chimaera ebf2     agaccgctctaatcacactcctcatttagacct-acacccgcacctgttc--agcaattaacatcacagattttgcatttaacat----------------tttgctgtgtttccaa---ttag-ttttatcgctcagta
chimaera ebf3     gggacattttattcatttacctcatttaaagcgcctttctgcacctgttc--aactattaatatcatgtaatttgtgcctaatgccgattttgct-taacattcattatatttctta---ttag-ctatatttccaaatg
lancelet          gggcacattggctagcccgttccatttgggtg--ttctctgcacctgtca--tgcaattagtgcgttataatgttcctgttgtgcccaatttggt--ggtgctcattatcttctgtaat-ttgg-attcattatgaaatg
velvet worm       gggagacttcattcatttaactcatttaaaggg-ttttccgcacctgtca--gatcataaataagccctaattttcacttaatgccaatttagct-taacagtcattattttctata---tcag-gt-------------
chiton            gtgagatttgattcattttctccattttgcgcgcctttcaacacctgttc--aggggtaaata-----------------------------------------------------------------------------

human EBF1        ------------------------------------------------------------------------------------------------------------
human EBF2        attaggtagggttattacagtgcatcaagcagggtcat-ggttattcccaagcctttaagcgctttccca-gatttgtttagggaagccactaagggcaaatggttga
human EBF3        attatgtatgattattac------------------------------------------------------------------------------------------
chimaera ebf1     ------------------------------------------------------------------------------------------------------------
chimaera ebf2     attagacgaggttattaccacatattaaactcggcaacaaagaattcccacggctttaatctttctctc---------------------------------------
chimaera ebf3     attatgtatgattattaccagaccctaggaacagtgatgggttattccctagacctttacatcttcttgtcaggctgttttcggaag-cagtgagaataaacatttga
lancelet          atcatacaggattgtgac------------------------------------------------------------------------------------------
velvet worm       ------------------------------------------------------------------------------------------------------------
chiton            ------------------------------------------------------------------------------------------------------------
```

Figure S17: *Coe* intron element

```
                                                                                                                                      CCAAT
human NR2F1         --------------------------------------------------------------------------------------------------------------ccaaccaatggcgtgcaggaggcttggcta
human NR2F2         ----------------------------------------------------------------------------------------------------t-gacggcg-------------------------------
chimaera nr2f1      ----------------------------------------------------------------------ttgtcaga-gcgtc----gtaggtaataaat-aagacgg-ctaaccaatggtgtgcaggaggc-tggcta
chimaera nr2f2      -------------------------------------------------------------------------------------------------aagt-gaggtgc-ctatccaatgcggtgcaaggggc-gggctg
lancelet            tgaccc-gaacaaagtggatttcctatgtaaacattcagcgcagggata-------------------tattgacaat-atgtc----cgagataataaac-ggcactg-ccagccaatgggatcagcgaggg-ctgcgc
acorn worm          tgaccc-ccataaagtggatttcctaagtaaacaacgagcgtaaggata-------------------tattgacaat-atggc----acccataagtaga-ggcagcg-acagccaatcagcgacgagctag-ctgcac
sea urchin          --------------------------------------------------------------------------------------------------------------------------------------------
centipede           tgacccagtgcaagtggcatgcgctgtgtaaacacaaagcgcaaggatatcggcgttggcagcgcggtcgccgacaat-atgtc-gcgctggataatgaacgggcggcg-tcagccaatgagagcaggccgag-tggtga
millipede           ----------------------------------------------------------------------ttgacaat-ctgtcggtagagcataataaac-gaggaaa-gcggccaatggcagagcaga----------
scorpion            --------------------------------------gcgcaaggatac--------------gtagtgccgacaat-atggc----ccggataac--ac-gccggcg-gcagccaatgagaagagggaaga-gcgttc
horseshoe crab      ----------------------------------------------------------------------------------------------------------------agccaatgagaaagcagttcg-accttt
sea spider          ----------------------------------------------------------------------------------------------------------------gcccactgtg------------------
velvet worm         tgaccc-cttcgagtggcatgcgctatgtaaacataaagcgcaaggatatcagc---------tatagtactgacaat-atgtc----taagataataaac-gacgcgt-ttagccaatgagagagcgcggag-gtgctc
cactus worm         --------------------------------------------------------------------------------------------------------------------------------------------
chiton              -------------------------------------------------------------------------------atgac----agggataatgaat-gatctca-gcgaccaataggagtttgtcgag-gcaggc
abalone             ------------------------------aacacggagcgtagggatatgtgc---------gggaggcctgacaatcgagga----gagtataatgaat-gatagtg-cggaccaataggattgcatccct-gagcgc
owl limpet          -------------------------------------------------------------------------------atgtc----ggttgtaatgaat-gacgtgg-agggccaatcagaacagtcgtaa-ctgtta
oyster              ------------------------------------------------------------------------------------------------------------------ccaataggagcttggaaga-tggtgc
nautilus            -------------------------------------------------------------------atattgacaattgtgct----ttgtataataaat-gatgctc-tagaccaatgggatcatatcaaa-ctgtgg
peanut worm         ---------------------------gtaaacactctgcgcatgaatatcccc------------agtattgacagctggatt----ctgggtaataaat-gagagag-cgagccaatgagcgcagggcggg-cccctc
sandworm            --------------------------------------------------------------------------------------------ataacgtat-ggcccag-ccgaccaataggagagctcgaga-ctgccg
Satsuma tubeworm     --------------------------------------------------------------------------------------------------------------------------------------------
shingle tubeworm    ---------------------------gtagatatacggcgtagtaa---------------------tattgacaag-------aaggctggttataaat-gttagct-tcagccaatgagaagcgagagtg-agagct
shamisen shell      ---------------tggatggtgtgtgtaaatactgcgcggaggaatatcggcat-------gaaattattgacaaacctcta----cgggttaacaaac-gatgcagtctagccaataggaatgtatgcta-gcgcgc
bootlace worm       --------------------------------------------------------------------tattgacagc-cagtg----tcggataataaat-gactcgg-gtagccaataggaagaggtggta-gtgttc
stony coral         --------------------------------------------------------------------------------------------------------------------------------------------
lace coral          --------------------------------------------------------------------------------------------------------------------------------------------
starlet sea anemone --------------------------------------------------------------------------------------------------------------------------------------------
pale anemone        --------------------------------------------------------------------------------------------------------------------------------------------

                                                                                                                                        CArG-box__     RFX______
human NR2F1         accttagcctcccattttctctccccccgattca-gggctctgaccagtca-gtcgcctttgattatcagttgccagcagcc----cttctcttggctccttgacacg----agctccatataaggca-gcgatctccat
human NR2F2         -----------------------------------agggggcggcgcg-cg-----------------------------------cgcgcctggccaggccaaccccggctgctgccttataaggcgcgccgtcgccat
chimaera nr2f1      acct--------tagcctccccagccccaattca-gggctatgac----ca-gtcgcctttgattatcagttgccaacagcc----cctctcttggctccttgacatg----agctccatataaggca-gcgatctccat
chimaera nr2f2      ga----------tgtcccagccccctcgaaccta-aagctctgac----ca-gtcgcctttgattgccagtcgccagcagccgccgctttggctccgtcactcgggtg---tagcgccatataaggaa-ggcatctccat
lancelet            gg---------cgtgacaggccccctttaccccc-gggctatgac----ca-gtcgcctttaattatcagttactaatagt-----ctccttgaacccccctgacatg---tcactccatatatggta-gtaattgtcat
acorn worm          cg---------aatgacaggccctgactaccctcaaggatagaag----ca-gtcgcctttaattatcagcttccaatcac-----ccccttgaacctagctgacatt---tctttccatataaggca-ataattgtcat
sea urchin          ----------------------------------------------------------tctaaacatcagtcaccaatata-----ccccacgaacttagctgacacc---gcctgccatataaggca-gtgggtgtcat
centipede           ac---------gcagtcatggcccg-atggctgg-ggggcacgac----ca-gtcgactctgattatcactcactaatagg-----cttcttgaacttaaatgacacg--ctcgcgccatatatggaa-gcagttgccat
millipede           ------------cagtcgtggccctctacctttagggggcgcgac----ca-ctcgcctttcattatcgttcactaatagg-----cttcttgaactcggatgacacg--------------------------------
scorpion            gc----------cagtcatgccctgattgcctag-ggggcatgac----cgtctagcccaggattatcactcgctaatagg-----cttcttgaactttaatgacacg---ttttgccatatatggaa-gtggttaccat
horseshoe crab      cg---------gcagttatgccctgattgcctgt-agggcacgac----cgccttgcctaggattatcactcgctaatagg-----cttcttgaactttaatgacacg---ttttaccatatatggaa-tacgttgccat
sea spider          -------------ccatgccccttgcctagcacg-aggccatgac----ca-gtcgcctccgattatcactgtataatagg-----attcttgaaccgaaatgacatg---tctatccttataaggaa---tatggtcgg
velvet worm         ac----------cgcccctgccccgcttgccggtgggggcatgac----ca-atcgcctctgattatcaaccgctaatagg-----ctgcttgaacttaaatgacatg---ttttaccatatatggag-ttagtatccat
cactus worm         -------------------gccctaattgcgcga-gggccacgac----ca-atgggctttaattatcacgcactaatgtg-----ctcgt-------------------------------------------------
chiton              ca----------tggctatgccccgcatgcctta-gggccgcgac----ca-tgcgacagttattgtcactcagtaatagg-----cttcttgaacctaaatgacaca---gtactccatatatggaa-gtagtcgccat
abalone             ta----------cggttatgccccgtgtggctgc-gggttctgac----ca-tacgacagtaattgtcactcaccaataca-----cttcttgaactcgcatgacacc---gctctccatataagga--gaggtaaccat
owl limpet          g-----------cggttatgccccgcgtggcgag-gggctttgac----ca-gacgacagcagttgtcactcaccgctaca-----cttcttcaaccttagtgactcc---cagcgccatataagga--ggtgtaaccat
oyster              gg-----------tgttatgcccctagagcgaag-gggcctcgac----ca-agcgactgccattgtcactcagcgatata-----cttcttgaacttatgtgacatt---agactccttatatggaa---aactgccat
nautilus            ta----------gtgctctgccctgcatggctga-gggctacgac----ca--acgacagtaattgtcactcactaatatg-----cttcttgaacccgaatggcagt---gtcatccatatatggg--cttgtatccat
peanut worm         tc---------acggaatggccgagactcgtgcgggggccatggc----ca-ctacttgggaattgtcactcgctcgcacg-----ccccccaacccccaatgacatc---acctgccttatatggca-gtggttgctat
sandworm            gg----------ctgccgtgccctccacccccc--aggccgtgac----ca-gtcgactgtaattgtcactctctaatagc-----cagcctcaacctaaatgacatg---ccctgccatataaggaa-gtggtttccat
Satsuma tubeworm     ----------------------------------------------------------------------------------------ccttgaacccaaatgacacg---gcgcgccatatatggaa-gacgtcaccat
shingle tubeworm    aa----------ctggcttggcccgtgtgtaga--cgtacagggc----ta-ctagtttcaagctgtcactccgtaatgac-----tggttacaaccttgctgacttg---tcctcccaaatatggaa-gaggtaaccat
shamisen shell      tt---------ctcagtcgctcattactggctct-cgtccacgac----tagtccgcctagaattgtcgctccgtaatggg-----ctctctgaacctaactgacacg---ttgtgccttataaggta-gatgttgtcat
bootlace worm       ag---------atgatctcgcctc--tcgccttg-aggccatgac----ca-ctggcttgtaattgtcactttctaatggg-----tttcttgaacctaactgacatg---cggagccttatatggca-cgagttactat
stony coral         ---------------------------------------------------------------------------------------------------cttgacagg----cttaccatatatagaa-tcagttgctag
lace coral          --------------------------------------------------------------------------------------ccttgaacctcgatttgacagc----cggaccatatatagca-gtggtttccac
starlet sea anemone --------------------------------------------------------------------------taatatta----tccttaacccttatatgacagt---tattgccatatatggtg-cacgcttccat
pale anemone        ------------------------------------------------------------------------------acta---ccctccttcccatatatgactct---tgctcccttatatggca-tg-gctgctat

                    _______     Prdm4________   NRRE__ NRRE__
human NR2F1         agaaacgtg---tcagtttcaatag-tagtgtcaaagttcactatatacaga-----------cattcgcgcagatcc-ccctttcggaaacattgctctgc-----
human NR2F2         ggcaacgtgcgctaagttgcagcag-tcgtgtcaaagttcactatatagagagctcagtgagctgatcgcggagaagccacttctg-------ccagccccggcgcc
chimaera nr2f1      agaaacgtg---tcagtttcaatag-tagtgtcaaagttcactatatacaga-----------cattcgcgcagatcc-ccctttcggaaaaattgctccgct----
chimaera nr2f2      agaaacgtg---tcagtttcaatag-tcgtgtcaaagttcactatatacagc-----------catccgcgcagatttcccctttcagaaaaattactccgcaagcc
lancelet            agaaacccg---acagtttcaacac-gagtgtcaaagttcagtatatacaga-----------catacaagcacatcctctcatca--aaaaattactccgctgtcc
acorn worm          agaaacccg---acagtttcaacaa-aagagtcaaagttcagtatatacaga-----------cattcacgcacatcc-cacttca--aaaaattactccgcact--
sea urchin          ggaaacgat---gcagtttgagcggctagtgtcaaagttcaca-tacacaga-----------caagagctcacgccc-ccgtttagctgcgattactccgctctgc
centipede           ggcgaccca---tcagtctcaacag-tcgtgtcatagttcaaaata-------------------------------------------------------------
millipede           ---------------------------------------------------------------------------------------------------------tc
scorpion            gccaacccg---acagtttcaacta-tcgtgtcatagttcaaaata-------------------------------------------------------------
horseshoe crab      gccaaccga---acagtttcaacta-ttatgtcatagttcagaatagctgaggttcagccacacgctc---------------------------------------
sea spider          tgcaacgcg---atagttccatcaga---------------------------------------------------------------------------------
velvet worm         ggcaacccg---acagtttcaacag-tagtgtcatagttcaaactagattgagtacatacagacattcacgaacatcccac--------------------------
cactus worm         -----------------------------------------------------------------------------------------------------------
chiton              agtaactcg---acagtttcaacag-ctgtgtcagagttcaaaacatataga-------------------------------------------------------
abalone             agaaactta---gcagtttcaacag-aagtgtcagagttca------------------------------------------------------------------
owl limpet          agaaactta---gcagtttgaaggg-agaagtcagagttcagt----------------------------------------------------------------
oyster              agaaactca---acagttttaatac-gaatgtcagagttcaaaacatacaga-------------------------------------------------------
nautilus            agaaactca---acagtttcaacag-cggtgtcagagttcaaaatagatagagtgctt-----cagacgggcacactctcccaaca---------------------
peanut worm         agaaactgg---acagtttcaacag-cagtgtcagagttcaaaacatatcga-------------------------------------------------------
sandworm            ggcaactgg---acagtttcgaggg-cggtgtcagagttcaaaacatactga-------------------------------------------------------
Satsuma tubeworm    agtaactgg---ccggttt-ggcaa-gagtgcc--------------------------------------------------------------------------
shingle tubeworm    ggtaacgtg---acagttccaactt-tacagtcgaagttcaaaacacataga-------------------------------------------------------
shamisen shell      agcgactcg---acagttttaacac-cgatgtcagagttcaaactata-----------------------------------------------------------
bootlace worm       agctgcacc---gcagtttcaaacagtcgtgtcagagttcaaaacatccagaga-----------------------------------------------------
stony coral         ggaaacccg---acatttccaacagattatgtcaaagttcagtcttttcaga-----------cat-----------------------------------------
lace coral          ggatacccg---acagttccaacagattatgtcaaagttcagcttttacaga-----------cat-----------------------------------------
starlet sea anemone ggtaacccg---acagtttcaacagcccatgtcaaagttcagttggttcaga-----------cat-----------------------------------------
pale anemone        ggagacccg---acagtttcaacagaccatgtcagagttcagagctttcaga-----------cat-----------------------------------------
```

Figure S18: *Nr2f* promoter

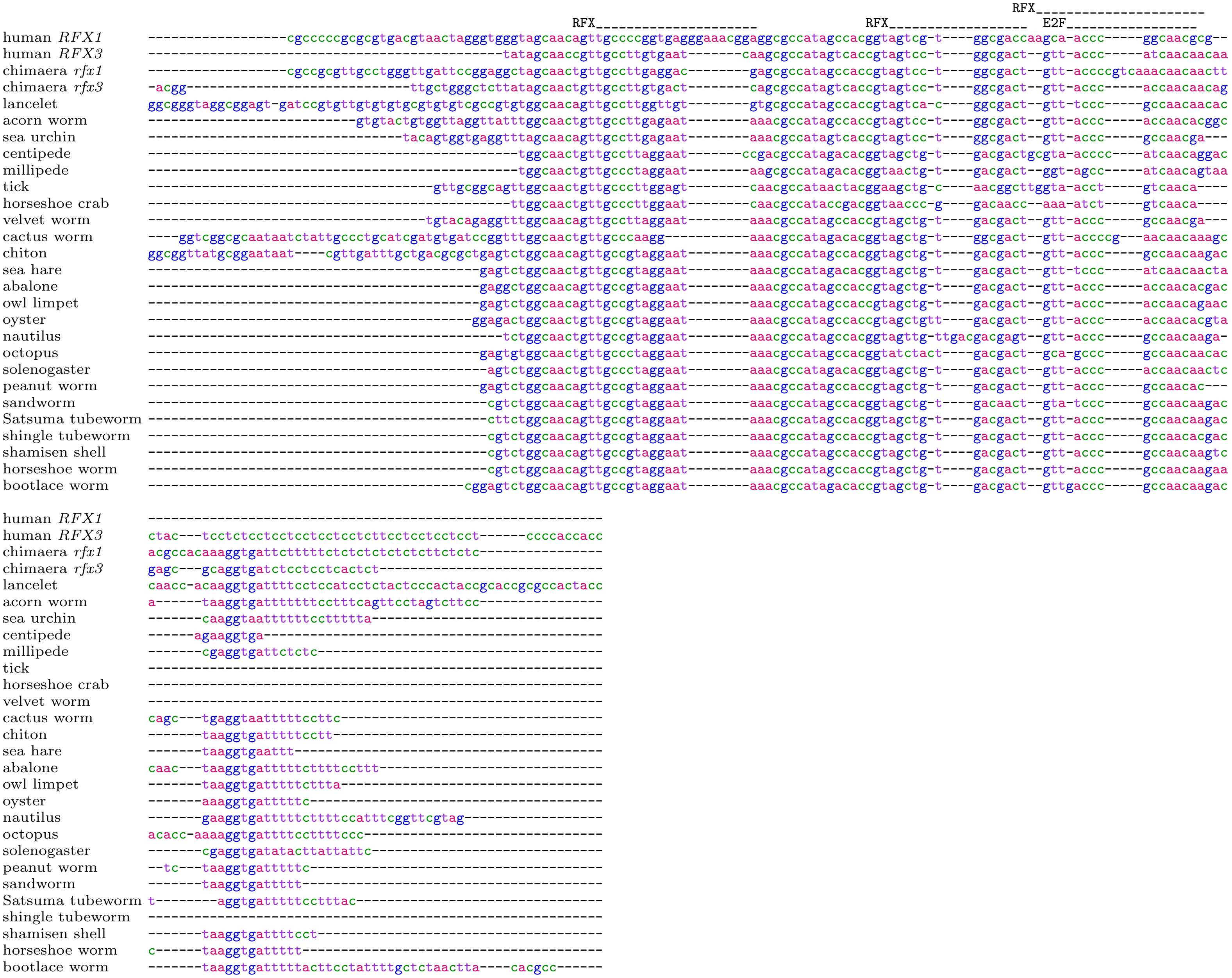

Figure S19: *Rfx1/3* promoter

```
                                                                     POU4______________
human SALL1    ---gctggcccggtgccaatcgcctttcaaagagcccctctatgattaatcgcaatgcattattg-at-aatcataattatag---------------------------------------------------------
human SALL4    --------------gccaatcagctgtcagggc------tcatgataaatcgcaatgcattattg-at-aataataattactg---------------------------------------------------------
chimaera sall1 ---gctggagagttgccaatcggctttcaagaagc----ggatgattaatcgcaatgcattattg-at-aatcataattatacccccaccccacccacccccaaaacccccccccccaccaccccaccaccaccacatgc
chimaera sall3 ----ctggagagcagccaatccgctgtcaggag---cggcgatgattaatcgcaatgcattattg-at-aatcataattatat---------------------------------------------------------
chimaera sall4 ----------------------------ggggggg----cgatgataaatcgcaatgcattattg-at-aatcataattacca---------------------------------------------------------
lancelet       --------------------------tcagcgcgctcctcgctgattaatcacaccccactattg-at-aatcataattatac---------------------------------------------------------
acorn worm     taatgagtagagcggccaatca------cggcaatgcattcgaaacataacgcaatgcattattg-ataaatcataaatttct---------------------------------------------------------
sea urchin     taattagtatatcgaccaatca------gaaaggaccattcatagcggagcaacaagctctattgcataaatcataaattggt---------------------------------------------------------
scorpion       ----------------------------------------------taatcgcaatgcattattg-at-aatcataatagtg----------------------------------------------------------
horseshoe crab ----------------------------------------------taatcttaatgcattattg-at-aatcataattgtg----------------------------------------------------------

human SALL1    -------------------------------------------------------------------------------------------caggacacatgcgcgctgcgccc--------------------------
human SALL4    ---------------------------------------------------------------------------------------------ggacatgcgcgttccggccgaaggggggta--------aatttccc-
chimaera sall1 acacacacacacacacacactcactcactcactcacacacatacacacacactcactcactcactcactcacacacatatatgcacaacgcacacacatgcgcactggaggcgccagg-gtcaatttccgtaattttgc-
chimaera sall3 -------------------------------------------------------------------------------------------ggtgacatgcgcactccacggccccgcagctaatttccgtaattttgc-
chimaera sall4 -------------------------------------------------------------------------------------------tcacacatgcgcactccactccccgca-----------gtaatttggc-
lancelet       ---------------------------------------------------------------------------------------------agacatgcgcaatgaccaccccttc--ttaatttccgtaattttcg-
acorn worm     ---------------------------------------------------------------------------------------------gtacatgcgcactggggcccccact--gtaatttccgtaattttgcc
sea urchin     ---------------------------------------------------------------------------------------------gtacatgcgcac-----------------------------------
scorpion       -----------------------------------------------------------------------------------------------acatgcgca------------------------------------
horseshoe crab -----------------------------------------------------------------------------------------------acatgcgcagt----------------------------------

human SALL1    -------------------------------------------------------------------------------------------------------------
human SALL4    -aactcca--ggaatttgt-----------------------------------------------------------------------------------------g
chimaera sall1 -aactcca---gaattagt---aattttttattcgc---------------ttgctgatgtatcaac------------------------------cacc-------a
chimaera sall3 -aactcgg--ggtatttgt--gaaatttttttttt-------ttttatttgttgctgatgtatctgc------------------------------cacc-------a
chimaera sall4 -aactccaccagaatttgtttgaaattttttttttttggggggttttatttgttactgatgcatctgc--catgtcgcgtcgcaagcagccgaacccccagc-------a
lancelet       -aactcga---gaatttca---aaatttttaatct-------ttttcattgttgctgctgctgtgggaggatgtcgcggcgcaagcaggcccggccgcaacatttggag
acorn worm     aaactcggc-------------gaatttctaatca--------tttccccgctgctgttgtttccag-ccatgtcgcgccgaaagcaggccaaaccgcagcacctaggg
sea urchin     -------------------------------------------------------------------------------------------------------------
scorpion       -------------------------------------------------------------------------------------------------------------
horseshoe crab -------------------------------------------------------------------------------------------------------------
```

Figure S20: *Sall* promoter

```
                                                                                CEBP__________                       E2F____________ Smad____    E-box_
human            ----------------------------aagagtt-----------aagaaacttgaa-accttgtttgcgcaacaatcag-------c----gcc---gcgg-agccgccaaagtgtctagac-tggcatatgatggga
chimaera         ----------------------------aagggtt-----------aagaaacttgag-accttgtttgcgcaaccatcgc-------tgcagatg---agag-ccgcgccaaaatgtctagaccggccacatgattgga
lancelet         ----------------------------------------------cggtaatgtgat-ctgttatttgcgcacccatcgg------------------cgga-gcgcgccaaaacgtctagac-gcgctgccgccgcac
acorn worm       actgtttttcggtgagatattc---cgggggagaagtaacagccaagtgtaatttgag-ctgttatttgcgcaacaatcat-------tttagact---cggg-gcgcgccaaaatgtctagac-gaccacgtgataatt
sea urchin       -------------------------------------------------------------gttatttgctcaacaatcat-------ttcagact---cgag-ccgcgccaaaatgtctagac-atccacatgacccaa
sea urchin       -------------------------------------------------------------gttatttgctcaacaatcat-------ttcagact---cgag-ccgcgccaaaatgtctagac-atccacatgacccaa
centipede        ----------------------------------------------cggtaattcgat-ttgttatttgcgcaacaatcac-------tgaagaca---tggg-gcgcgccaaaaagtctagacgaatcacgtgtccgct
millipede        attctttttcgcgaaaatggcgagaggagaagcatataatcagtagccgtaatttgat-attttatttgcgctacaatcgt-------ccgagacag-tgtgc-gcgcgccattgagtctagac-gatcacgtgat----
scorpion         -----------------------------------------------agtaatttgat-ctcttatttgcgcaacaatcgt-------ttcgg-----------gcgcgccgaaaagtctagac-gtccacgtgatcggt
tick             -------------------------------------------cgaaggtaatttgat-cggttatttgcgcaactatctcgcgcagacagaagga---ggag-gcgcgccgtcctgtctagac-gggcacgtg------
horseshoe crab   --------------------------------------------------------------ttatttgcgcaatgatcac-------cttaaaca---ccga-acgcgccaaaatgtctagac-actcgtgtgagcata
horseshoe crab   --------------------------gggaggaacgtagtctcagaaagtaacttgat-cacttatttgcgcaacaatcgc-------atcagaca---cta--ccgcgtcaaaatgtctagac-acgcacgtgaacata
horseshoe crab   -------------------------------------------------taatttgat-ctattatttgcgtaacaatcgt-------atctgtgc---ttag-ccgcgccaaaatgtctagac----------------
horseshoe crab   -----------------------------------------------agtaatttgag-ctcttatttgcgcaacaatcgc-------ttcagaca---cca--gcgcgccaaaatgtctagac-gtgtacgtaata---
velvet worm      --------------------------gggaggaatgtaatcattggaagtaatttgat-atgttatttgcgcaacaatcac-------tttagaca---tggg-gcgcgccaaaaagtctagac-aagcacgtgactaac
cactus worm      --------------------------------------------------------------------------cagtcac-------tatagatg---tgtg-gcgcgccagcgagtctagac-ggccacgtga-----
abalone          ----------------------------------------------cagtaatttgat-atgttatttgcgcaacaatcat-------agtcgaca---tcag-ccgcgccaaaaagtctagac-acccacgtgctacat
nautilus         ------------------------------------------------gtaatttgat-atgttatttgcgcaacaatcac-------tttaggca---tcagtccgcgccaaaaagtctagac-tgccacgtgaccgtc
octopus          -----------------------------------------------tgaaatttgatcccgatatttgtgcaacaatcact------gaaagacagagtgag-ccgcgccaaagtgtctagac-tagcacgtgacccgg
peanut worm      -----------------------------------------------agaaacttgat-atgttatttgtgcaacaatcac-------tgtagaca---gtgg-ccgcgccatggagtctagacgagccatgtgaccatt
sandworm         ------------------------------------------------gaaacttgat-acgatatttgcgcaacaatcac-------tttagaca---gcga-ccgcgccaaaaagtctagacaaggcatgtgactgaa
Satsuma tubeworm --------------------------------------------------aactcgat-gccttattggcgcaacaataac-------tttagaca---gcta-ccgcgccaaaatgtctagacctcgcacgtgacccgc
shamisen shell   -----------------------------------------------agtaattcgat-aggttatttgcgcaacaatcac-------tttagaca---tcag-ccgcgccaaaaagtctagacaaaccatgtgatcaag
stony coral      ---------------------------------------------------attgaaa-ttggtatttacgtaacaccctc-------atcagacaactatag-acgcgcctggctgtctagac-aagcatgtgacagga
lace coral       ---------------------------------------------------attgaaa-ttgttatttgcgtaaca-ccac-------atcagacaactatag-acgcgccaatgcgtctagac-atgcacgtgacaata
```

```
                       CCAAT
human            ggcag-ccaatgactccgcggcgctcc---------------------------
chimaera         gccaa-ccaatgactcaaaagtgtccc---------------------------
lancelet         gga---ccaatcagcgcgcagct-------------------------------
acorn worm       ttgaa-ccaatcaagtgttagcaatgg-tgtgg-cagtaagtgttgtaaatttc
sea urchin       gtcaa-ccaatcagcgtagaagtaatcgtgtggtcagagagagtagtaaatttc
sea urchin       gtcaa-ccaatcagcgtagaagtaatcgtgtggtcagagagagtagtaaatttc
centipede        cccgaaccaatggcggcgc-----------------------------------
millipede        ------------------------------------------------------
scorpion         tatag-ccaacc------------------------------------------
tick             ------------------------------------------------------
horseshoe crab   ctcag-ccaatggtt---------------------------------------
horseshoe crab   ttcaa-ccaatagc----------------------------------------
horseshoe crab   ------------------------------------------------------
horseshoe crab   ------------------------------------------------------
velvet worm      ataaa-ccaatcagatct------------------------------------
cactus worm      ------------------------------------------------------
abalone          cttgg-ccaatcagcgtgcagct-------------------------------
nautilus         gtcgg-ccaatca-----------------------------------------
octopus          ccgcc-tcaaccaatcaga-----------------------------------
peanut worm      tccga-ccaatca-----------------------------------------
sandworm         attgg-ccaatggc----------------------------------------
Satsuma tubeworm cccag-ccaat-------------------------------------------
shamisen shell   ataag-ccaatca-----------------------------------------
stony coral      atcaa-ccaatcaa----------------------------------------
lace coral       tttca-gccaat------------------------------------------
```

Figure S21: *Smad6* promoter. This matches four places in the horseshoe crab genome: three upstream of genes annotated as "smad6-like", and one upstream of the edge of a genome fragment.

```
                                                                                                                         HOX___________
human        -------------------------------------------cttcaagt---agcccccattgaatagac--------taagttgacactgcgtgacagtgaaacaacataataaaaaatacatgagc--------cc
chimaera     -------------------------------------------cttcaagt---agcccttattgaatagac--------taagttgacactgcatgacagtgaaacaagataat-aaaaatacatgagc--------ca
lancelet     tgaatgggttcgccgctgtaggcctccattgttcccgcttgttctgcgtat---taattatacaaagggggc--------tgcagtgacacaagaggcaaaaataacaacataataaaaaatacatgagc--------cg
acorn worm   tgaatggaccggttactctatgcggccattgttccgccttgttc-gggcat---taatgttataaaagagtt--------tgctgtggcacaagaggcaaaaataacaacataataaaaaatacatgggc--------ct
sea urchin   -------------------------ctattgtccaagcttgttcagcgtat---taattatataaaggacccgtggtttggggaggtttgcagaacggaaaaataacaacacaatcaaaaatacatgaacgccttcaata
stony coral  ------------------------------------------------------taattatagaaaagaa-----------acagaaagacaaggaaaacgaataacaacacaataaaaaatacatgagg--------ag
lace coral   -------------cgctgtgcgtcgttattgtaaatcttactttcgcatattgataattatataaaagaa-----------actgagagacaagggccgagaataacaacataataaaaaatacatgagg--------tg
pale anemone ------------------------------------------------------taattatataagagaa----------tataggtgaacaagaggcgagaataacaacataatcaaaaatacatgagg--------tg
```

```
                           MEIS___________
                              FOX___________                           SOX_______                                          SOX_______
human        ctgaataggagcaggcgcataaataaataaaatgggtgaccaaaactggataaactgaatgacaaaacggtgaaaggggaacaaaaagatatttaacacgctagattagcattagaatgcgatctacaaggcaga--aca
chimaera     ctgaatagaagcaggcgcataaataaat-aaatgggtgaccaaaactggataaactgaataacaaaacggtgaaaggagaacaaaaagatatttaacacgccggattagcattagaatgcaatctacaaggcaga--aca
lancelet     ctgaatgcccgaactgacataaataaat-aaatgagcgatcaactgtagataaactgaataacaaagat-tgaatggcgaacaatgag---tataatgcgactgatctacatgacaaaagaatatacatactgga--aca
acorn worm   gtgaatagctgatatgacataaataaat-aaatgagcaattcacacaaagggtgttgaatagactagtt-tgaatgacatacaatagg---tgtaacgctgctaatttgcatgacaatagggcatacaacttagt--aca
sea urchin   gcgaagggtgcgcatgacataaataaat-aaatgag---------------------------------------------------------------------------------------------------------
stony coral  ttgaatgaaatagatgacataaataaat-aaatcgacgatcaagaagagataaaatgagtaacaaaagt--gagtgaattacaattgc---tctaa-actgac-atctccataacaatggattaca--------------
lace coral   ttgaatgaaagagatgacataaataaat-aaatggacgatcaagaagagataaaatgaataacaaaggt--gaatgaattacaattgc---tctaatactaat-atctccataacaaaagattacatccacaaaaataca
pale anemone atggatgaaagcgatgacataaataaat-aaatagacgatcaagaggagataaagtgaataacaatagt-ggaatgaattacaattgt---cttaatactcct-atcaccataacaatgacatacatcaacaacgc-aca
```

```
human        attga-tgaatag-gtttaccggc--caagaaagaaatggactaaat-gccctttgaatagatatgcttt----------
chimaera     attga-tgaatag-gtttaccggc--cgagaaagaaatggactaaat-gccttctgaatggatgtgcttt----------
lancelet     aaggc-tgaatag-aacgaccggg--gtataaataaatagacccaaa-caggcttgagaagaatggcgtgaatagaaagg
acorn worm   aaggcgtgaaggg-atcgaggggga-gagtaaataaatagaggcaaa-cagcattgaattggtggctttgaatagagagg
sea urchin   --------------------------------------------------------------------------------
stony coral  --------------------------------------------------------------------------------
lace coral   ataac-tgaatag--------------------------------------------------------------------
pale anemone ataag-tgaatagcggcgacagaaagatctctgtaaataaaccaaaaccaactacgataggaatgg--------------
```

Figure S22: *Sox21* enhancer

```
                                                               WRE__             CCAAT                                                  GC-box_
human SP5          -----------------gggtc-tccaggcggcaa--ggccccctttgat---caggaaaatccaattatttgtgtatat--acatttcccacatagtgattacttcattaattgtcccgcctttta-tctcctcccttcc
chimaera sp5       -----------------gggtc-tcctggcggcagc-tgaaggctttgat---cagacaaatccaattatttgtgtatat--acatttcccacatagtgattacttcattaattgtcccgcctttta-tctcctcctttgc
chimaera sp5-like  --------------------tc-ctgtgggagtggccggggccctttgat---gaagtcaacccaattatttctgtacatggagatctcccagatggtgataacttcttcagt----------------------------
lancelet           -------------------------------------------ctttgatcaagagttggcccctatttagcatgtagat--agattacgtggctagtgattaggtcgttcttttacccgcctttt--gtcccgcccaaga
acorn worm         agtggcgtccaatcagtggaca---gtttggtcggtgacacgtctttgat---ggcacgaatccaatttgcagtgtgtct--aaaagtggcacatagtgattacttcatttacttgcccgcctttt-gtcccgccttgcc
sea urchin         -----------------------cttggcggtccgt-cggcacctttgat---aactgaaatccaattatttgtttatac--aaaagtggcagtgggctattacttcattaagttgcccgccccattgtaccgcctatcc
horseshoe crab     -----------------------------------tggcaccccctcaaa---ggtcggaatccaattatttgtgtg-gc--taatgctagaagtagtgattac----------tttccctcccct---acctgccctact
velvet worm        ---------------------------------------------------------------------------------------------------------------------ccctcccaa---tccggcctccct
cactus worm        ---------------------------------------------------------------------------------------------------------------------ccgccccat---gcctgcccccct
chiton             --------------------------------------------------------------------------------------------------------------------ccccgcctcg---ttcggcccatct
sea hare           -----------------------------------------------------------------------------------------------------ttgc---------ttccccgccctc---tccgtcccctcg
abalone            -------------------------------------------------------------------------------------------------------------------gtcccgccttc---gcctgcccctca
oyster             ----------------tgaatg---ggttccttggtattgaagctttgat---ctcctcaatccaattatttatgtgtgt--aaacgtcctgcgagaagattac----------ttgcccgccctc---gacaaccccgtt
nautilus           ----gcagccaatcgccgcacg---ccgccctcggcgcttctcctttgat---ctcccgaatccaattactgcgctgtat--acgttctctacgaggtgattgc----------ttgcccgccttc---gccaacccctct
peanut worm        ------------------------------------ggcctgtctttgat---gtgccaaatccaattatttgtgtatat--aaattgcacatgggacgattag----------ttccccacccct---gccggcccctcc
shingle tubeworm   ----gccctcaatagtcaaacaaccccctcagcggcacttttgctttgaa---gttgaaaatccaattatttgtgtgtgt--aaattccctgcctagtgattag----------ttgccctccctt---gccagcccctgt
shamisen shell     -----------------------------ggtcggcgtcggtcctttgat---ctcgtaaatccaattatttgtgtatat--aaattccctacgaggtgattag----------ttgcccaccctt---gccagcccctct
horseshoe worm     ---------------------------------------------------------------------------------------------------------------------------------------------
bootlace worm      ---------------------------------------------------------------------------------------------------------------------------------------------
stony coral        agcgatggccaatcaatgagca---ctttgctcggc-ggcttgctttgat---catagaaatccaat--------------------------------------------------------------------------

                                                                                 HOX_____               Fox______
human SP5          catcccccct----------------------------------------------------aataatcag-ttcttttatccagaccaaca-aacacaccataggagctttgtggatt---caaa-ggatttgctttcgc
chimaera sp5       catccccatccccatccccccatccatcccccacccccccacccccatcccccccctataataataatcagccccttttatacataccaaca-aacacaccacaggagctttgtgtatt---caaagggatttggttcccc
chimaera sp5-like  ---------------------------------------------------------------------------------------------------------------------------------------------
lancelet           caacccctct----------------------------------------------------aataatcgc-tagtattctacatgtcaaca-aacaagccgcgggagccctgtgtata---gaag-ggattgaatttc-c
acorn worm         ctgcccacgt----------------------------------------------------aataatcgc-ttctattatatgtgtcaaca-aacacacagcaggccctttgtcagca---caaa-agattcgatttc-c
sea urchin         cggcccctct----------------------------------------------------tataatgaa-cgctattttgcgagtcaaca-aaccctcttgcgggtcttttgtggtgaactaat-ggacggggcccc-c
horseshoe crab     aataacctcg----------------------------------------------------aataatccg-caccattataagtgtcaaca-a-----------------------------------------------
velvet worm        aataactg------------------------------------------------------cataatcag-cgccattataagtgtcaaca-aacactttcggagacgcgaggggata---taaa-agatttgatttc-c
cactus worm        aataatggcg----------------------------------------------------cataatcaa-cgctattatgattgtcaaca-aacactcgccagcgagcatatggaca---taaa-agattgaatttc-c
chiton             aatatctccg----------------------------------------------------cataatcaa-acctattatatgagtcaaca-aacagcctctaagaagcttgtagaca---gaaa-ggatttaatttc-c
sea hare           gacaacagcg---------------------------------------------------tcttaatcag-ctctatttttcgtgtcaacacagtccgctctaagaaacatatggaca---aaaa-ggatttcatttc-c
abalone            aataacaaac----------------------------------------------------cataatcag-ccctattatgcgtgtcaaca-cactcgctctaagaagcatatggact---gaaa-ggatttaatttc-c
oyster             aataacaagt----------------------------------------------------tataatcgc-ttctattatgactgtaaaca-atctctctcttagaactttgtatttt---gaaa-ggatttaatttc-c
nautilus           aataacaccg----------------------------------------------------tataatcag-cgctattataagtgtcaaca-aacactctccgaccgccatatggaca--taaaa-aggttgaatttc-c
peanut worm        aataacgccc----------------------------------------------------cataatcag-ggctattatgtctgtcaaca-aacacgctcaaggaagcctatgtgct---cgag-ggatttaatttc-c
shingle tubeworm   aataacaagg----------------------------------------------------aataatcac-ttctattatgactgtcaaca-aacacagccttacttctatatggaca---caat-ggattaaatttc-c
shamisen shell     aataacattg---------------------------------------------------gcataatcag-ctctattataaacggcaaca-acccctcgccaggaagcatatggaca---taaa-ggatttaattct-c
horseshoe worm     ---------------------------------------------------------------ataatcag-tgccattatcaacgctaaca-aacactctcttagactcctatggaca---gaaa-agatttaatttc-c
bootlace worm      -----cgcgg----------------------------------------------------cataatgga-cgctattattcccgtcaaca-aacacacactgcgaccactgccaggc---ccac--tgttcaatatc-c
stony coral        ---------------------------------------------------------------------------------------------------------------------------------------------

                                               CCAAT                 WRE__                   CCAAT
human SP5          ttctgaaag-agccgctattctttgatgattgggtagc--ggcaaacttcaaagcca-taaatcttccctctgactggctggcggcccagcaaagtcctt-----------------------------------------
chimaera sp5       ctctgaaag-agccgctggtctttgatgattgggtggtggggggaacatcaaagtggctgtggctctctgctcactggctggtgggctgg---------------------------------------------------
chimaera sp5-like  ---------------------------------------------------------------------------------------------------------------------------------------------
lancelet           ttttaaatc-gcccgctgatcacatatgaatggcgggc--ggcacacatcacaggaa-ttttctcgcgtgccgattggctggcggtgagatcagaggcagttgc-----------aagtctggtctgtgt-taccgccttt
acorn worm         ttttaaatc-acccgctagttggcgctaattggacagc--ggtaaagatgacacgaa-gtgccctccaatgcgattggttggcggtgtttcaaagctcctcgtt------------cgcgtctgatgtgc-gaccgcctat
sea urchin         tttcaaatc-caccg-caccgcttcctcattggtgggc--ggcatctctcaaaggac-ttagaccgtcagatcattgggtagcggcgagataaaagactagacg-----------taatgcggctttccctcactgactat
horseshoe crab     ---------------------------------------------------------------------------------------------------------------------------------------------
velvet worm        tttacattc-acccgcctttgctaacggattggcggtc--ggcgaacttcaaaaatc-cgcgagctatttacaattggctcgcggtctgtgaaagcaatagctc-------------gttcacatagtgt-taccgcctct
cactus worm        ttttaaata-acccgccagcccactctcattggtctgc--ggcactatcaaacaggc-atctctcgt-tcgatattggctggcggcacgtcaaagagagcgaagcgccgcatatttatgtatgaagtgtgcaccgcctat
chiton             ttttaaacc-acccgcctattcaatgtaattggctggc--ggtagacctcaaagggc-tggaggcgcgctccgattggctggcaccacatcaaagc---------------------------------------------
sea hare           tt-------------------------------------------------------------------------------------------------------------------------------------------
abalone            ttttaaacc-accccgctattgatcgtaattggctggc--ggcgaacatcaaaggaa-aaatatcgcgttttgattggagggcggtagatcagagccgatactc-gca-----------gcactaagtgt-gaccgcctat
oyster             ttttaaactaacccgcctatagatttcaattggctcgc--ggcaaacttcaaagaca-ttgtcaagtattttgattggctcgcggaaacatcagag---------------------------------------------
nautilus           ctttaaacc-acccgcgtgccggctgtaattggctgtc--ggcgcacttcaaaggag-cgctcctgcgccccgattggcgggcggtggatctgaggcgatggc--------------------------------------
peanut worm        tttcatttc-tgccgactattgattgtaattggtccgc--ggtggggatcaaagctg-gtgcctcgcgcgctgattggctagcggcacttctaagacccaggtt-------------------------------------
shingle tubeworm   ttttaaatc-gcccgcctattgattttgattggatagc--ggcagacatcaaaggaa-aaccccaac-ataggattggatatcggtacttcaaaggggatcaac------------tgttcgtaaagcat-tacggcctat
shamisen shell     ctttagtgc-acccgacagcgtgagactattggttggc--ggtatacatcaaaataa--ctcctagagctccgattggctggcggcatcttcagatctccgtga-----------taacgcttgaagtgt-tactgcttat
horseshoe worm     tttcaaatg-acccgcttctaacattttattggatagc--ggtacgattaaaggcaa---acattcactcgtgattggttagcgg--------------------------------------------------------
bootlace worm      gcc--aacc-aaaccaccattgatcctaattggcccgc--ggcggacatcaaagaca-ccgcattctcctctgattggtcagcggcacatcaaaggccattg---------------------------------------
stony coral        ---------------------------------------------------------------------------------------------------------------------------------------------

human SP5          -------
chimaera sp5       -------
chimaera sp5-like  -------
lancelet           tcacac-
acorn worm         tcacact
sea urchin         acacac-
horseshoe crab     -------
velvet worm        tcgc---
cactus worm        tgaca--
chiton             -------
sea hare           -------
abalone            tcacagt
oyster             -------
nautilus           -------
peanut worm        -------
shingle tubeworm   tgatgtt
shamisen shell     tga----
horseshoe worm     -------
bootlace worm      -------
stony coral        -------
```

Figure S23: *Sp5* promoter

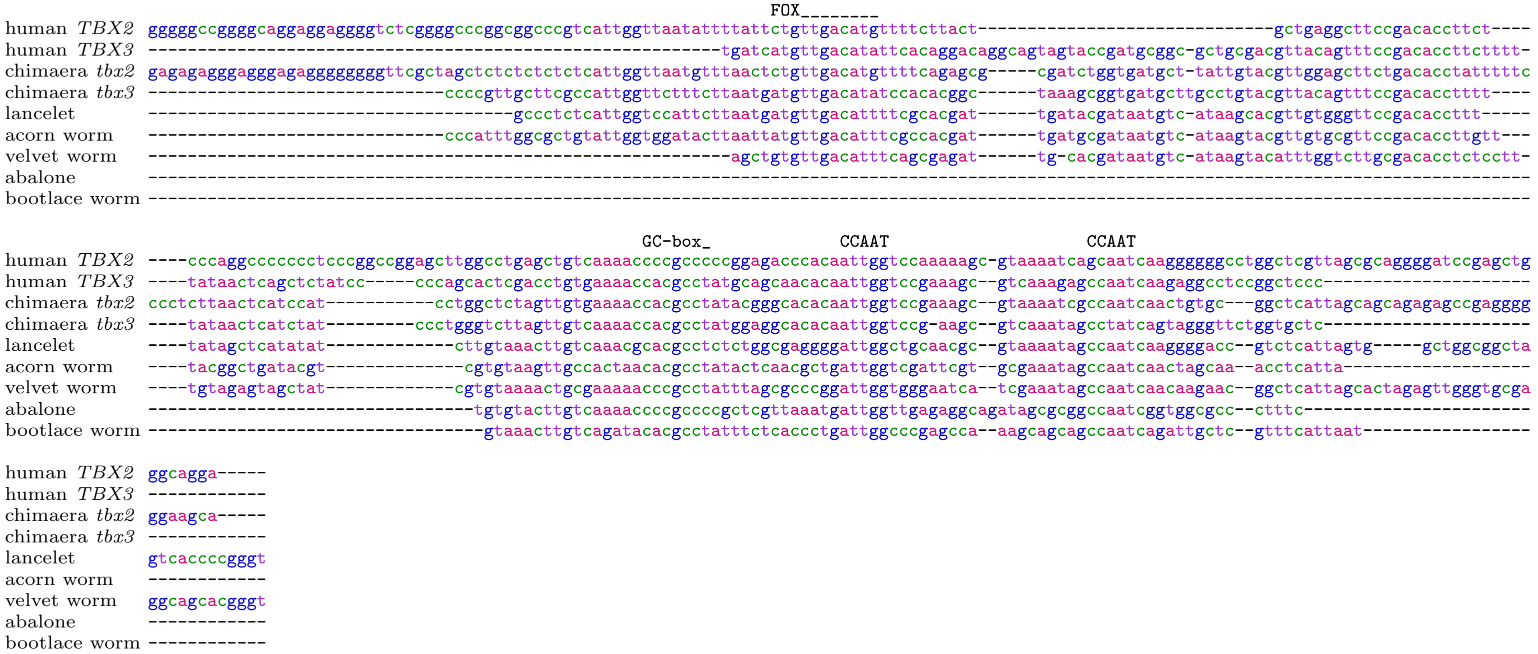

Figure S24: *Tbx2/3* promoter

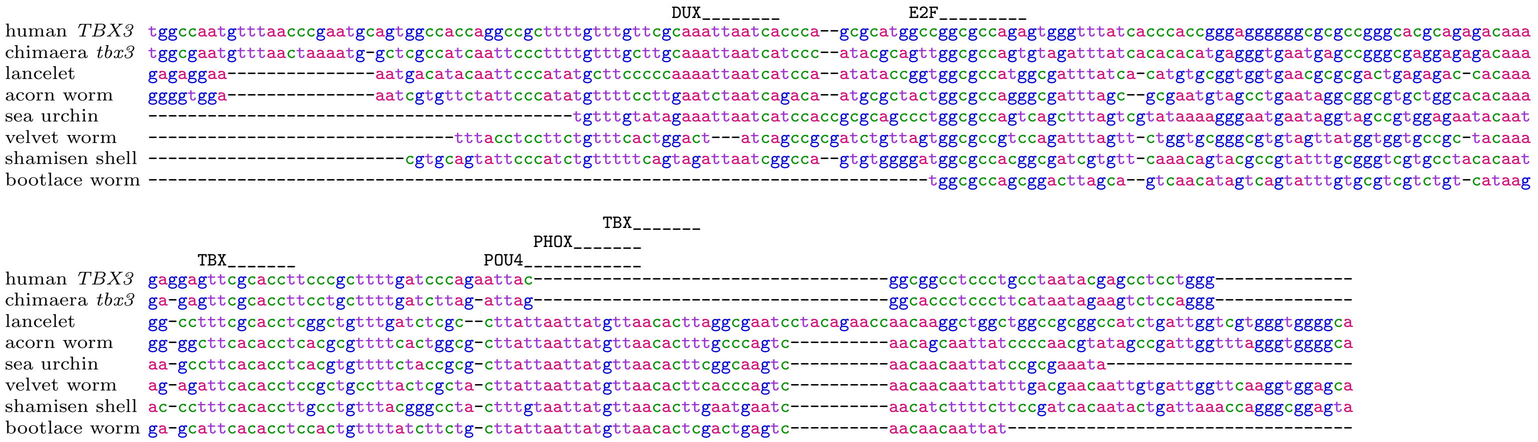

Figure S25: *Tbx3* enhancer

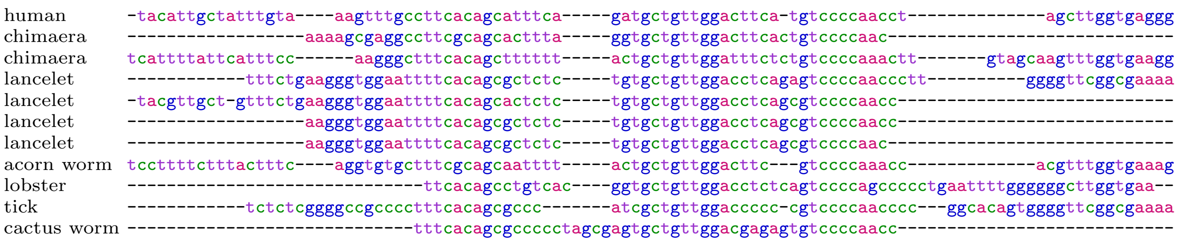

Figure S26: *Znf207* 3′-UTR. This matches two nearby places in the chimaera genome downstream of *znf207*, and four nearby places in lancelet downstream of *Znf207*.

```
                                                                                                                                            miR-92_
                                 miR-130                                                                                                         Let-7__
human CPEB2    ccattttgacatttgttatgcactatttttatatctctgtgagagatttttccaacagtcagc--tatttta-------------------tggcacactttttttgactgatgacatctc-ctttgctatacctcaa--
human CPEB3    -----------tttgttatgcactactactttttgtat-tctagacagttttcaaaagttggcagatttttttttttgtttttaaggaaaaaggcatgc--tttctgactgacatgatc---ttttgcaatacctcaa--
human CPEB4    --------atatttgttatgcactacttttgtatatct------tgtttttccaacagt-gaa--cattttt-------------------aggcacact-tttcactgacgggatatctctttatgcaatacctcaa--
chimaera cpeb2 ccatattggcatttgttgtgcactacttttgtatcttggtgagagttttttccaacagtcagc--tgtttta-------------------gggcacac--tttttgactgatggcatctc-ctttgcaatacctcaa--
chimaera cpeb3 --------acatttgttatgcactactttttgta-----tctggacggttttcaacagtcagctgattttt-------------------aagcgtgc--tttctgactgacatgatc---ttttgcaatacctcaa--
chimaera cpeb4 --------acatttgttatgcactacttttgtatatct------tgtttttccaacagt-gga--tattttt-------------------cagatcact-tttttcacagatggcatctctttttgcaatacctcgatt
lancelet       -----------------------------------------------------------------acttttt-------------------aggcaggc--tttccaccta--tgtacc---atttgcaatacctcaagg
cactus worm    --------------------------------------------------------------------------------------------------------------------------------------------
shamisen shell --------------------------------------------------------------------------------------------------------------------------------------------

human CPEB2    -tttttggaatttagagaagaaatcagtagttttgc--aatgttaattatttagatattt--------------------------------------------------------------------------------
human CPEB3    --ttttgaaatataggaaagaaaatgatattttctaagtaagtttattc---------agaaaaatatatattaatttaattctgca-agaaaatgattta------acagatgggta------actttatttttttcat
human CPEB4    -tttttcatattgcaaagagtagc------tttttg--tacttttattactgagagatcttcatatac-------ttcattttttaa-tataaataatttt-----aataaattttat------tttcttatattctgct
chimaera cpeb2 -ttttctgaatttagaagagaaatcgatagttttgc--tatgtttattattgaaagatctgcatatata-attaatttaattcagca-aataaatgattta-----acaaaattgata------tttttatttttcatac
chimaera cpeb3 --ttttgaaatataggaaagaaattgatattttctaagtaagtttattc----------taaaaatatatattaatttaattctgca-aggaaatgattta------acagatgggta------attttatttttttcat
chimaera cpeb4 ttttttcatattctaaagagagatgaatagtttttc--tatgcttattgttcaaagatctgcaaatatg-gttaacttaattgtgca-aagaaatgagttg-----aagcaatcagtt------ttgctttctttcgttt
lancelet       ttttccagaattt---gagcaaatagatatttt-----gaagtttattcttgaaagttaggaatgagagtattaatttaattctgca-----gatgatttaaagagaagatgtgggta------attttatttttcttgt
cactus worm    ------------------------------------------------------------ggagtgacattattatttaattctgca-atcaaacagttgaaaaataaaatatgggtaagattgattttatttttctcgt
shamisen shell -------------------------------------------------------------aagatgtatgttaatttaattcagcacacaagataatttatggtaggaaaatgggta------attttatttttt----

human CPEB2    ----------------------------------------------------------------------------------------------------
human CPEB3    gct------------------tatttgtgtttt-----ttgaaaa-------tgaagaagttagagctttataactataatattaaagatcatatctaac
human CPEB4    ttt------------------tatacattt----------------------------------------------------------------------
chimaera cpeb2 atc------------------ttgctttct----------aaatt-------tggaagggtctaaacatttgta--------------------------
chimaera cpeb3 gct------------------tatttgcttttt-----tggaaat-------tgaaggagctagagctttataactttaatatggagaattgtagttagc
chimaera cpeb4 tat------------------tattttttt----------------------------------------------------------------------
lancelet       att------------------gttctgctttttgcatatgaaaac-------tggaaagctttaaacttttttattata---------------------
cactus worm    aatattattatgtatgattccttttcagcctttgcctgtaaggatgccagtgcaggagattttaaacttttttattata---------------------
shamisen shell ----------------------------------------------------------------------------------------------------
```

Figure S27: *Cpeb2/3/4* 3′-UTR. Previous studies showed regulation by miR-92 [13, 14] and miR-130 [15], and predicted these Let-7 and miR-92 binding sites [13].

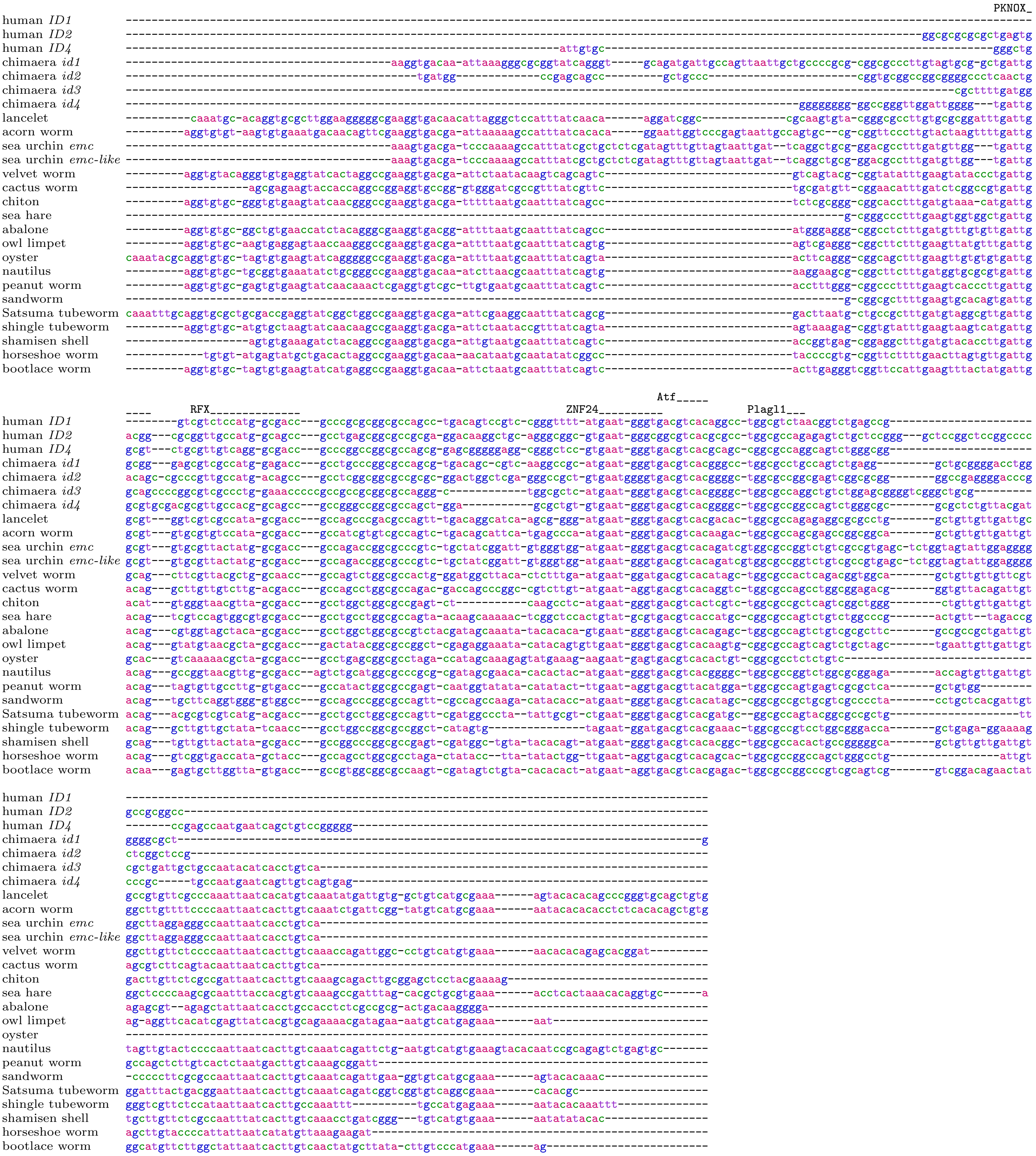

Figure S28: *Id* enhancer

```
                                                                                            E2F___________
                              MEIS____               CCAAT                               HOX_______            HOX_______     E-box_
human LIN28B         --------------------------------------------------cccttaa-ggatacgaggtgaaattagtagcaagaaagcatgtaattgacaaagtcacgtgtgctcagggggccagaaactggagagagg
chimaera lin28b      ttatgtgcggtgacaggttggagcagagtgggattggctcggcgctgcctctccgaa-ggctgcgatgtgtaattagcggcaagaaagcatgtaattgacggagtcacgtgcgccag---ggccaggaa----aggagga
chimaera lin28b-like ttatgtgcggtgacaggttggagcagagtgggattggctcggcgctgcctctccgaa-ggctgcgatgtgtaattagcggcaagaaagcatgtaattgacggagtcacgtgcgccag-----------------------
lancelet             ttattctcaatgacaggtagcagagccacgctattggctgttgccagggcggccgct-gattggtatgtgtaattagtggcaaagagccatgtaattg--tacgtcacgtgtagcag-----------------------
velvet worm          --------------------------------attggttgttgctaagataataattccactagtatttgtaattagtgccaaagaagcatgtaattg--tacgtcacgtg-----------------------------

human LIN28B         agagaaaaaaatcaaaagaaggaaagcacattag
chimaera lin28b      aaaaaaaaggggctaaagaggaaaagcacgttag
chimaera lin28b-like ----------------------------------
lancelet             ----------------------------------
velvet worm          ----------------------------------
```

Figure S29: *Lin28b* promoter

```
                                                                                                         MRE_____
human MEX3B      ------------tgctttctaac-----------atttgtataaatag-aat--tcagtgcaagttacaaaaattctgttgcaccac-tctagtttt-agtatttcta-ttttaata--catttgtttaccacttgttta
human MEX3C      ----------------------------------------------------------------------------------atcaa-tatagttta-gtatttgctaattttacta---cactct-------tttgtta
human MEX3D      ---------------------------------------------------------------------------------------------ttta-attttttttt-ttttttac---ttttacttttta-cctcttg
chimaera mex3b   aaggaacagttaagctttctaac-----------atttgtacaaatagaaaa--ccagtggaaattac--aaattctgttgcaccac-tatagttta--gtatttcta-ttttaata--catttgtttaccacttgttta
chimaera mex3c   ccg----------------------------------------------------------------------------------------------------------------------------------tttgtta
chimaera mex3d   --------------------------------------------------------------------------------gcaccag-tacagattactaaatattca-agtttatc---atttgtttacct-cttactg
lancelet         ------------tgctttctaac-----------atttgtataaaata-gct--acacatgccgatacgataatttcgttgcac----catagtctaggcatgtacta-ttttaata---tattatttacaa-cagttta
acorn worm       ----------------ttctaacatat-tatattatttgtataaatag------caactgactatatggtaaaatccgttgcact---catagctag-aatttacttatttttaata---tattattta----cagttta
velvet worm      ----------------------------------------------------------------------------------------ataggttta-gtattttcta-ttttaata---cattatttacca-cagttac
cactus worm      ----------------------------------atttgtata-----------gtagcgaccgacttgtatgatttgttgcacat--gttagttta--gtatttcta-ttttaata---tattatttacta-ccgtttg
chiton           ----------------------------------atttgtataaatag-ca----tttcaaaatgaccatttttgttgtttcacact-catagttta-gtattttcta-ttttaatatgatattatttaataccagccga
abalone          ---gaagatttaagctttctaacatacatatattatttgtataaatag----------attctcaaagacttaatttgttgcacatc-catagttta-gtattttcta-ttttaata-----ttatttacta-ctctgtg
owl limpet       -----------------------------------------------------------------------------gttgcactta-aatagttta-gatttttcta-ttttaata--ttattatttactatttgactg
nautilus         aaagcaagattatgctttctaac------attatatttgtataaatag-------------tatctgaaaaaaatttgttgcacaac-catagacta-gtattttcta-ttttaata-----ttatttacgaccagtctg
octopus          --------------------------------------------------------------------------tttgttgcacaacttagagacta-gtattttcta-ttttaata------tatttacgagctatctg
Satsuma tubeworm --------------------------------------------------------------------------------------------------------------------------------------------
shamisen shell   ---------ttatgctttctaatg----catattatttgtatgaatag-------caaaactgacaacaattaatttgttgcacaac-catagttta-gtattttcta-ttttaata--ttattatttatcacaattctg
pale anemone     a--------------------------------tatttgtacaagata-tgtagtcataatttggatacgatattccgttgcactac-tatagttgg-------acta-ttttaata---t-------------------

                                                                              MRDE_________________________________
human MEX3B      tgtatatgtaggtgatgttacttgagcttaaatgtacttta--------ctgagcaaagtttaaaaaaca---aagta-----tattttatttt----atgataaag-ggcctttaacct--catggtcaaat--actaa
human MEX3C      tgtatatgtagggaagtcatagggattataaattcaattt-----------gagtaaaatttaaaa-----------------ccatatatttt----atgataaag-ggcctttaacttaagatggccaaag-cactga
human MEX3D      tgtatatgtagggaatttata---gggaaatatgtacttta--------tggaataaattttaagaacta---aaata-----tattttattttaaataaagtaatg-gacctttaatcttacacagctaaat-tactga
chimaera mex3b   tgtatatgtaggtaaagttacttgagcgtaaatgtacttta--------ttgagcaaagttt-aaaaacg---aaata-----tattttatttt----atgataaag-ggcctttaacct--catggtcaaat--actaa
chimaera mex3c   tgtatgtgtagggaagtgataaggattataaattcaacctattgagtcgaaacaaaaagtttaaaa-----------------ccatatatttt----atgataaaatggcctttaacttaacgtggccaaag-cactga
chimaera mex3d   tgtatatgtagggaatttata-ggactgaaaatgtacttta-------tttgaataaattttaaaaaaaac-taaata-----tattttatttt----aagataatg-gacctttaaccttacatggctaaag-tactga
lancelet         tgtatatctaggtgtgaaaca-tgaccgcaaatatactatg-------gagaaaaagtttactaaataca---aaata-----ttttttatttt----gggcctggt-aaccatg-------tttggccagatatactaa
acorn worm       tgtatatgtaggtgtgagata-tgaccaaaaatg---------------ttgaacaaagtttaatacaaatgcaaatat----ttttttatttttgtttggccagct---------tactgaaatggccagatatactaa
velvet worm      tgtgtatattagtatagaact-ttatcaccagca---------------aaaaaatgaatactaaaga---------------tattttatttt-ataaggtccatt-----------tcgttgtggccat---tactaa
cactus worm      tgtatatgtaggtgtga---------------------------------------------------------------------------------------------------------------------------
chiton           tgtgtatatgtagagaaactc---aatacagatgttgtaaa--------caagtgaaaagtctaaaaaaa---agata-----ttttttatttt---caaggccaat-gcaaatg----t--tgtggccagat--actaa
abalone          tatat-tgtagatgttccaca--gattaccaataactcgtc-----------gacaaagtacaagaa------agata------tttttatttt----gaggccaag-caaaata---------tggccgtt---actaa
owl limpet       tatatgattgtagacgtttat----------------------------aaagacaaaataccatataag---atgaaaagatatttttattttaataaagaccact-aaataaa---------tggtcgat---actaa
nautilus         tgtatatgtaga---------------------------------------------------------------------------------------------------------------------------acaag
octopus          tgtatatgtaga---------------------------------------------------------------------------------------------------------------------------acaag
Satsuma tubeworm -------------------------------------------------------------------------------------ttttatttt----atggcaata-tgcagtg---------tggccaaa---actaa
shamisen shell   tgtatgtaggtgtgtgactatggaacccaaagtaaagatta--------agatgcagagtttaaagttaa---atacaaaga-tattttatttt-gttaggctcagc-taataag----------agcctgta--actaa
pale anemone     --------------------------------------------------------------------------------------------------------------------------------------------

                    miR-374
human MEX3B      ---tattatatttgctgagaca---agatttgaaa------------ttgtatcaagagttttatttttctgacattta---------------aagttctacataataaaggtaaaact
human MEX3C      ta-ttatatatttgctgtaaag------------------------agaattataagagttttatttttctgatattaa-aagttacttaataaagacttgtttccattaacttgaa---
human MEX3D      ---ttatatatttgctgagctgatttaagggttaa-------aaaaattgtatcaagagttttattttttgacttcaaa--gccttcttaataaagcctcttttctacatgt--------
chimaera mex3b   ---tattatatttgctgagaca---agaattgaaa------------ttgtatcaagagttttatttttctgacattta---------------aagttctacataataaaggtaaaact
chimaera mex3c   ta-ttatatatttgctgtaaag------------------------agaattataagagttttatttttctgatattaaaaagttacttaataaagac-cgtttccattaaagtgaa---
chimaera mex3d   ta----tatatttgctgaactgacttgatggtatg---------aaattgtattaagagttttattttttgacttaaaaaaaccttcttaataaagactttttacagtatgt--------
lancelet         tattattatatttgttgagact--tgaggattata------------aaattgtaagagttttatttttctgccattaa---------------agttgtattacaataaatgacaagct
acorn worm       ---tattatatttgccgagatt---tgagattatt----atggaattgttattaaaaagttttatttttctgtcattaa---------------agtaaaagttct--------------
velvet worm      ---tattatataaactaagaga-----------------------tatacaatttaaagttttatttttctgccagta------------------------------------------
cactus worm      ------------------------------------------------------------------------------------------------------------------------
chiton           ---tattatatttgctgaactc---aaatattata-------------aatgtaaaaggttttatttttctggcattaa---------------aa------------------------
abalone          ---tattatatttgctgaaact---cagaagtata-------------agatgtaagagttttatttttctgccattaa---------------agtcaagttccaa-------------
owl limpet       tattattatatttgccggaact---caattataag-------------agatgtaaga-ttttatttttctgccattaa---------------agtaaa--------------------
nautilus         t-----------------------------------------------------------------------------------------------------------------------
octopus          t-----------------------------------------------------------------------------------------------------------------------
Satsuma tubeworm tgttattatatttgctggaccc---agaattttaa-------------gattgtaagagttttatttttctgccattaa---------------aaat----------------------
shamisen shell   ---tattatatttgtggagagt---aagaaaaaaagtataaaaaaaagaattgtaagagttttatttttctggcattaata-----------aaagttgtatttcaa-------------
pale anemone     ------------------------------------------------------------------------------------------------------------------------
```

Figure S30: *Mex3* 3′-UTR. MRDE: previously predicted Mex3b-responsive destabilizing element [16]. MRE: resembles a MEX-3–recognition element [17]. miR-374: previously predicted miRNA binding site [18].

```
                                                           GLI___________                  RFX______________                                             CC
human PTCH1    gaggctgccc-----cgaagccgaggatgcacacactgggttgcctacctgggtggtctc-tctac-tttggtgagctgttgctaagcagctaataatag---tcatgtttgctgagtaatttctgccct-ccgcgagcc
chimaera ptch1 --------------------------------tggatgcactcacagcctgggtggtctc-tctac-tttggtgagctgttgctaagcagctaataatag---tcgtgtttgctgagtaatttctgcct--tcctcggcc
chimaera ptch2 --------------------cccacccccccattgatgctctggcagtctgggtggtcctggccac-tttggtgagctgttgcttagcagctaataatagt--agatgtttgctaagtaatttgcttcttccctttagcc
lancelet       gaaggcgcctggcggtggaggatggatgtgtcagggtacccctacgacttgtgtggtctg-tcttc-tttggcgagccgtttcctagcagcaaatgacggg--ccgtgttactcgagcaaattgtggag--cccgcggcc
acorn worm     ----------------------tcctctttttatcctatcccaacttcttgtgtggtcta-cctctttttctggtgacgttactaagcaactaatgagaac--tcgtgttacatgagtcatttggccg---tgagtgacc
scorpion       ---------------------------cctctttggtggctaggcaacgtgtgtggtccggacgac-gtcggtgatccgttgcctagcaactaat---------------------------------------------
horseshoe crab -----------------------------gccacagcgtccccgcgacgtgtgtggtcttctccac-tttagcgaaccgttgcttggcaactaatgcaaggttcgctgttacgagaggagctagaaag---ccacgagcc
horseshoe crab --------------------cccgcattctcaacagcgtccccgcgatgtgtgtggtcttctatac-tttagtaagccgttgcttggcaactaatacaaggctcgctattacgagaggagctagaaag---ccacgagcc
chiton         -------------------------ttccctcttcgacctaccgcaatctgggtggtctt-tctac-tttggtgagccgttgcctggcaactaatgtgcagc-tgatgttacttgagcaatctgcgcta--taattgacc
sea hare       -----------------------------------------tcacttcttgggtggtcta-tctac-ttgggtgagctgttgctaagcaactaatgttgggc-tggtgttacacgagcaatcctactga--taattgacc
abalone        --------------------------------tgggttcccttccttcttgggtggtctg--ctac-ctgggggagctgttgccaggcaactaatgttgggc-tggtgttacacgagcaatcctactga--taattgacc
oyster         --------------------cttatcgcactgttggcggtcccgcaacttgggtggccgagcttctatgtggtgaacggttgcttagcaactaatg--------------------------------------------
shamisen shell -----------------------------ccgtttgttcctggccagcttgggtggtctt-tctac-tttggtgagctgttgctaggcaactaatgtcgggc-tgcagttacatgagctgcctgtgggc--ttaagaacc

               AAT
human PTCH1    aatcgcgtcgcagaaacccaagccatctgaca------------gccccgggggcggga----g
chimaera ptch1 aatgagatcccagcaatacaagccttctcccagccatctgacg-gccctggaggcgggtttt-g
chimaera ptch2 aatgggaatccggtcgagaggactttccatcaactctatctgatgggctggaggcgggtt----
lancelet       aatcagagcgcgggaaggtgacccctcgatcacgggagctcga-ggcggagaggcgggttttgt
acorn worm     aatcagattgaagaaaattgacccttctgtggggcc-gtttga-caggagggggcgtggtt---
scorpion       ----------------------------------------------------------------
horseshoe crab aatcgaagcg------------------------------------------------------
horseshoe crab aatcgcagcg------------------------------------------------------
chiton         aatcagagcgctgatctgtttcccatcaatcatg------------------------------
sea hare       aatcag----------------------------------------------------------
abalone        aatcag----------------------------------------------------------
oyster         ----------------------------------------------------------------
shamisen shell aatcatcgccgcgctctgcgcgccatcaatcacgtc-accgca-ttcccgggggcgggccttgt
```

Figure S31: *Ptch* promoter

```
                                                                                                                  RFX___________ NRRE_____
human             --------------aagggggcggagctttggcg------------------tggggcggccaatagt------------gggggt--ggctgcgtgggtcgccatggggacgggg---ctgttcccggggaggctgtg-
chimaera          --------------aacggggcggcgccggctgc------------------ggcgcgggccaatgag------------ggcggc--ccgtcgtttggtcgccatggtgacgggg---ttgttcccggga----cctg-
lancelet          ----gtgggttttgaaaattatagcttcaagtatgggctgtctcccgccaaacaaatggaccaatcagcgaagaccttttgacaag--tcatcatcgtgtttccatggttaccggg---tcgggctgggg-----cttg-
acorn worm        tcacgtgatgcaatgctaagttagtttctagatc--tccataccaattactgcgaactagccaatcaggatacactcttcgacgggtcatcaactgcagtttctatggtaacgggg---tcggactcggc-----cttg-
sea urchin        ----------------------agtttctagaat--ccctgaattaat-cagtgcttcgaccaatcaacgtcgcgtactgggcagt--tcacgatgtagtcgtcatagctacgggg---tcagtctctgc-----cttg-
centipede         --------------------------------tc--cccttagcaacc-acaccgaacgaccaatgagcgtgcgggctttgacagg---tccattgacgtttctatggtaaccggg---tcagtctcaac-----cttg-
millipede         -----------------------------------------------------------------------------------------caccacaatgtttctatagtaacggagtttttggtgtcgtc-----cttg-
scorpion          ----------------------------------------------------------------------------------------atcaattaatgtttctatggtaacaggg---tcggtgtcgtc-----ctga-
scorpion          ------------------------------------------------------------ccaatcacatagccgcatttgacaga-tcccaattgatgttactatggcaacaggg---tcggtgtcgtc-----ctga-
tick              ------------------------------------------------------------ccaatcacaaggcagtctttgacag---atcaattgatgtttctatggtaacatgg---tcggtgtcgtc-----ctga-
horseshoe crab    ------------------------------------------------------------ccaatcacgttactgcctctgacag---atcaattgacgtttctatggtaacaagg---tcggtatcgtc-----gtat-
horseshoe crab    ----------------------------------------------------------------------------------------atgaactgatgtttctatggtaacaagg---acaaaatcatc-----gtga-
horseshoe crab    ----------------------------------------------------------------------------------------atgaactaatgtttctatggtaacaagg---agaaaatcatc-----gtga-
horseshoe crab    ------------------------------------------------------------ccaatcacacagctgcttcccacag---atcaattgatgtttctatggtaacgagg---tcggtgtcgtc-----gtgt-
velvet worm       -------------------------------------------------------atgggccaatcacatttgagcttttgacag---gtcgattgatgttgtcatggaaacaggg---tcggtgtcgtc-----cttg-
chiton            ---------------------------------c--cccacaactactataattaaacatccaatcgcaatgtgccatttgacagg--tctacttgacgttgtcacggtaacgagg---tcagtgtcgtc-----cttg-
sea hare          ------------------------------------------------------------ccaatcaacgcgaagcgactgacagc--cgcagttgatgttggcaagacaacgggg---tcagtgtcgtc-----cttg-
abalone           ------------------------------------------------------aactatccaatagagacttgccatttgacagg--tctacttgatgttggcaaggcaacgggg---tcagtgtcgtc-----cttg-
owl limpet        ------------------------------------------------------aaccgaccaatcgagactagctatttgacagg--tctacttgatgttggcaaggcaacgggg---tcagtgtcgtc-----cctg-
oyster            ------------------agttggtttctagatc--gcagattctgctataattaacgaactaatcgtagaacgcctttcttcagt--tctacttgtggttgctatagtgacgggg---tcagtgtcgtc-----cttg-
nautilus          -cacatgatgttagaccggaccgttttaaggcac--tccaaacttactataattaactacccaatcgtaagttgcattttgacagg--tctacttgatgttggcaaggcaacgggg---tcagtgtcgtc-----cttg-
octopus           ------------------------------------------------------aactacccaatcgtaacttgccttttgacagc--tctccttgacgttggcaaggcaacgggg---tcagtgtcgtc-----cttg-
peanut worm       ----------------------------------------------------------------------------------------acccagtgacgtacccatggaaacgagg---tcagtgtcgtc-----cttg-
sandworm          -------------------------------------------cctactcagcggctcagccaatcggagcgcttctcatgacag---ttctcttgatgtcgtcatggtgacgagg---tcagtgtcgtc-----cttg-
Satsuma tubeworm  tcatgtgatattttgactcatgagtttctagtctc-cccacatctactaaaattactccaccaatcgtattgctccgtttgacaag-tctcattagatgtcgccatggtgatgagg---tcagtgtcatc-----cttg-
shingle tubeworm  -----------------------------------------------------------------------------ttcgacagg--tctttctgacgtcgctatggaaacaagg---tcagtgtcgtc-----cgtg-
shamisen shell    ----gtgatgtttgaacacgacagtttcaaggct--caccaatcctccgtaataactcgcccaatccaagattgctatttgacagg--tccttgtgacgtctctatgacgacgagg---tcagtgtcgtc-----cttga
horseshoe worm    ----------------------------------------------------------------------------------catg--tggtgatgacgtcactatagcaacaagg---tcagtgtcatc-----ctta-
bootlace worm     ---------------------------------------------------------tacccaatcgaaaggcgctatttgacagg--tcttcttgacgtcaccatagagacgggg---tcagtgtcgtc---ctcttg-

                                 PKNOX_______
                          MEIS____
                           TBX______________
                              E-box_
human             --at-gggttgacaggtg-cgtgacagt-------------
chimaera          --at-ggcttgacaggtg-cgtgacaatccagctcttca--
lancelet          --at-gggttgacaggtg-cgtgacagtccagctatccagc
acorn worm        --at-ggcttgacaggtg-cgtgacagttcagctacccagc
sea urchin        --at-tgcttgacaggtg-cgtgtcagtatagctacccagc
centipede         --atgggcttgacaggttgtctgacatttc-----------
millipede         --at-ggcttgacaggtg-cgtgacagttc-----------
scorpion          --at-gtcttgacaggtg-cgtgacagtttaacttgacagc
scorpion          --at-gtcttgacaggtg-cgtgacagtttaacttcacagc
tick              --at-gtcttgacaggtg-tgtgactgttcaaatccacag-
horseshoe crab    --at-gtcttgacaggtg-cgtgacagttcagcctcacagc
horseshoe crab    --at-gtcttgacaggtg-tgtgacagtttaac--------
horseshoe crab    --at-gtcttgacaggtg-tgtgacagttcaac--------
horseshoe crab    --at-gtcttgtcaggtg-cgtgacacttcggccacacag-
velvet worm       --at-ggcttgacaggtg-cgtgacagttcagctccacagc
chiton            --at-ggcttgacaggtg-catgacagttcagct-------
sea hare          --at-ggcttgacaggtg-cgtgacagttcagctct-----
abalone           --at-ggcttgacaggtg-cgtgacagttcagctctc----
owl limpet        --at-gtcttgacaggtg-cctgcgagt-------------
oyster            --at-atgttgacaggtg-cgtgacagttcagctccc----
nautilus          --at-ggcttgacaggtg-cgtgacagttcagct-------
octopus           --at-ggcttgacaggtgccgtgacacttcagctac-----
peanut worm       --at-ggcttgacaggtg-cgtgacaatccaact-------
sandworm          --at-ggcttgacaggtg-cgtgacagtttcagctc-----
Satsuma tubeworm  --at-ggcttgacaggtg-cgtgacagttcagc--------
shingle tubeworm  --aa-gcattgacaggtg-catgacagttcagctgc-----
shamisen shell    tcat-ggtttgacaggtg-cgtgacagtcagctccc-----
horseshoe worm    --at-gggttgacaggtg-cgtgacagttcggc--------
bootlace worm     --at-ggcttgacaggtg-cgtgacagttcagct-------
```

Figure S32: *Ssbp2* promoter. This matches two places in the scorpion genome and four places in horseshoe crab, all upstream of genes annotated as “Ssbp3-like”.

```
human ZNF503           -----------------------------------------------------------------------------------------------------------------------ctcttttcccttcaagtcgca
chimaera znf503        --------------------------------------cgtccgccttttgttcgcatacaaattgagtttactctgagaacgatttatttccctatattgaatgcctcttttcaaa-agccgttctttttcaagtcaca
lancelet               aaaggggcacaaattgccgcctacttacag----tgtttcggcgttctttgttcgc-cgccaaacgggtttattt--ccaacgattta-tatcaccaattcaatgacgcttttcaaagcctttcttttttttcaaatcaca
acorn worm             ----------------------------------------gactccctttgttgtcggacaaattgagctcacac--agaccgatttactttcggcaattcactggccttttcagaa------aactttttcaaattacc
sea urchin znf503      --------------------------------------cggtctctttgttgtcacacattaattgcgattatt---gggccgatttatctcggggaatcataccccctttcaaaag------ccgtttttcaaattact
sea urchin znf503-like --------------------------------------cggtctctttgttgtcacacattaattgcgattatt---gggccgatttatctcggggaatcataccccctttcaaaag------ccgtttttcaaattact
millipede              --------------------------------------------------------------------------------------------------------------------------------------------
scorpion               --------------------------------------------------------------------------------------------------------------------------------------------
scorpion               --------------------------------------------------------------------------------------------------------------------------------------------
scorpion               --------------------------------------------------------------------------------------------------------------------------------------------
tick                   --------------------------------------------------------------------------------------------------------------------------------------------
horseshoe crab         ------------------------------------------------------------------------------------------------------------------------------------------cg
horseshoe crab         --------------------------------------------------------------------------------------------------------------------------------------------
velvet worm            ---------------------------------------------------------cgcaaattgcgtttattt--tgaacgattag-tgtgaccaattcaatgtgtttt------------attctttatttgctcgg
cactus worm            --------------------------------------------------------------------------------------------------------------------------------------------
abalone                --------------------------------------------------------------------------------------------------------------------------------------------
shamisen shell         agacggatacaaacttccatct-cctacaggatcggctttcaaattctttgttcacgcacaaattgcgtatactt--tcagcgattag-aattaccaattccgtgtggttt------------atcctttatttgcctct

                                                       MEIS__________
                                           MafF_____________         PKNOX___________          MEIS__________                  NFI_____________
human ZNF503           cccgc-cttg---taatcagcaacaaaa-gctgacataaatcacgggaggattgacaagagctgaca-aataactgattgattg-cggtcgaggaacattgacaattgatggaggggtggagatgcccaaagaagggggg
chimaera znf503        ccagg-cttg---taatcagcaacaaaa-gctgacataaatca-acgcagattgaca-gggctgaca-aataactgattgattg-cggtcggggaa-attgacagtctggggaggggaagg-------aggggaggggag
lancelet               ccggaccatg---taatcaacaacaaaa-ggtgacatgaatca-gccaggattgaca-gccttgaca-ggagattgattgatgg-gggtctggat--atcgacggatggcgacatgccagg--tgattaa----------
acorn worm             ctagc-catg---taatcgcctacaaag-ggtgacataaatca-gacgcgattgaca-gccatcacg-ttggaatgattgatta-cgct---------------------------------------------------
sea urchin znf503      cgagc-catg---ttatcaacaacaaaacgctgacataaatca-gacggaattgaca-aggctgacaccgcatttgattgatcg-cag----------------------------------------------------
sea urchin znf503-like cgagc-catg---ttatcaacaacaaaacgctgacataaatca-gacggaattgaca-aggctgacaccgcatttgattgatcg-cag----------------------------------------------------
millipede              -----------------cgacaacaaaa-ggtgacataaatca-agtatgattgacgtgccgtgaca-ggcgcttgatggatga-ggtcct-------------------------------------------------
scorpion               -------------------acaacaaaa-gctgacataaatca-accttgattggca-cttgtgaca-ggtgtgtgatagatgg-cgctgtaaac--catgacggctggcggcttgccaag--tcattag----------
scorpion               -------------------acaacaaaa-gctgacataaatca-accttgattggca-cttgtgaca-ggtgtgtgatagatgg-cgctgtaaac--catgacggctggcggcttgccaag--tcattag----------
scorpion               ----------------ttgacaacaaaa-gctgacataaatca-actgtgattggca-cttgtgaca-gccgcttgatggatcg-ccttgtagcc--tgcgacgcctggcatcttgccaag--tcatta-----------
tick                   -------------------acaacaaaa-gctgacataaatca-tgcctgattggca-gctctgaca-acgggctgatggatag-cgttgtaaaa--gttgacagctggccccacgccaga--tcatta-----------
horseshoe crab         tcgcactgta---ggcccgacaacacaa-cctgacataaatca-actgtgattgaca-cttctgaca-gatccttgatggatgg-tgttgcaaag--actgacaactggcggcttgccaaa--tcatta-----------
horseshoe crab         ----------------tcaagcactaag-cctaacataaatca-gttcctactgaca-ctcttgact-ctcgactgatggatga-cgttgtaaac--cttgacagctggcactttgccaag-------------------
velvet worm            ctatataatgtatacattaacaacaaaa-ggtgacataaatca-acgttgattgaca-gagctgaca-ggcgattgattgatcg-tagtctgaag--cgtgacggctggcggcttgccaag--ctatta-----------
cactus worm            ---------------attactaacaaag-ggtgacataaatca-gttgtgattgaca-gccgacgca-ttgaagtgattgatgg-cg-----------------------------------------------------
abalone                ---------------attaacagcgcgc-ggtgacataaatca-agcttgattgacg-gacaggcca-tcaaaatgattgattgttggggcatga--ggtgacgtctggctagctgccaag--taatta-----------
shamisen shell         cttca-agtgtatacacgaagaactaaa-gctgacataaatca-agcatgattgacg-tgcggctca-tcaacttgattgatca-cagtcggtgc--aa-----------------------------------------

human ZNF503           gagggaggaaa
chimaera znf503        ggcagtggaaa
lancelet               -----aatgtc
acorn worm             -----------
sea urchin znf503      -----------
sea urchin znf503-like -----------
millipede              -----------
scorpion               -----tacatc
scorpion               -----tacatc
scorpion               -----------
tick                   -----------
horseshoe crab         -----------
horseshoe crab         -----------
velvet worm            -----------
cactus worm            -----------
abalone                -----------
shamisen shell         -----------
```

Figure S33: *Znf503* enhancer. This matches three places in the scorpion genome, two upstream of genes annotated as "Noc-like", and two places in horseshoe crab upstream of Noc-like annotations. (*noc* is an insect homolog of *Znf503/703*.)

```
                                                                     GC-box_
                                                             EGR_____________                                                  DUX__________             ALX____
human ZNF503     gg---cctgacctgtcaggcggatta-----tcttcgagggagattaataggggaggcgggctgcaataataatcgttttgtttgatgtgacaactctgatagg-cgttgatttacttacaaactgataggcttttaatt
human ZNF703     tga-ccctgagccgtcaagaag-----------gaacagccagattaatggggtgggcgggctggggtaataatcg-tttgtttgatgtgacaagcctgatagg-cgttgatttacttacagactgatgggcttttaatt
chimaera znf503  ga---cctgacctgtcaagcggatta-----tcatacagagagattaatagggtgggcgggttgcaataataatcgttttgtttgatgtgacaagtctgatagg-cgttgatttacttacaaactgataggcttttaatt
chimaera znf703  tgaccccgagactgtcaagcga-----------agtcccggagattaatggggtgggcgggttaaagcaataatta-tttgtttgatgtgacaggcttgatggg-cattgatttagttgcggactgataggcttttaatt
lancelet         ta----taaacgtgtcagtaggattaccgaatgatggagagtgataaatggggtgggcggtctatgataataatcattttgtttgatctgacacttttgatagg-cgttgatttactttaaaactgataggcttttaatt
acorn worm       -------------------------------------------ataaatagtatgggcgtacatgggtaataatcttattgtccacgttgacggttttgattga-cgttagtttact---------acgtcgttttcata
velvet worm      ta----taaacgcgtcagccacttcactgattgatgcaagcagataaataggataggcggggcagccaaacaatcattctgtttgatttgacacgtctgatagaccgttaatttact-----------------------
shamisen shell   ------------------------------------caagtagattgatggagaaggcggggtgtctaaacagttcccttgtaccacttgacacgtctgatagg-gcttaatttacttaaagcct--ctgcgttccgatt

                 ---
human ZNF503     gagcgcctccg-ccgagcccgagatgaaagggaagcggcg----------------------------------------gtg
human ZNF703     gagcacgccat-cctagtc--acttcaaag------------------------------------------------------
chimaera znf503  gagcgcctacatccaagttttagatgaaaggaaagcggcg----------------------------------------gcg
chimaera znf703  gagaacctccaattccgaggcacatcaaag-----------------------------------------------------
lancelet         gagcgcctaag-gagcggc-gacttcaaacaagtcgaggacg--taagctgtctcttttggcggggcaacaaaacaatttccc
acorn worm       gcacaccgccg--agcggc-gacttcaaagaaatactccccactatagctgtct---------gagcaacaaaacaatttgcc
velvet worm      -----------------------------------------------------------------------------------
shamisen shell   actatcaaaacacggcggccaacatcaaaggcgtcctgcgtagcctaccgtctcgtttgggcaagggaacgtaacaatttgcc
```

Figure S34: *Znf503/703* enhancer

```
human       caggttggcagatctggagggctaatccaaacaa----ggcgcttgacttgtgaggtc-gcgagtctagacagagctggtggctcggcggctccttatctttccctggaatcagccgaagag
chimaera    caggttggtgtatctctgagcctaatcaaaacaa-------gtttgacttgtgaggtcctcacgtctagacagagctggactctcggcggctccttatctgagaacggaatcagcagagagg
acorn worm  ----------------------aatcaaaacagttttgtttttttgacttgtgaggtctgtacgtctagacagaaagcgtcgcaaggcggcggcttatctcccttcggcatcaaactaa---
```

Figure S35: *Isl1* enhancer

```
human       gtctttaatgtaccattaactattgtctttacacaatatgggaactgt---aaagcatgacatgtgttataataaaacaca-ttttcaatg-atacacttggacttgagg-ctggggtgc-agcaaacaaaca
chimaera    gtctttaatgtaccattaactattgtctttgcacaatatgggaactgt---aaagcatgacatgtgttataataaaacaca-ttttcaatt-atacacttgtactggaag-ctctgatgcaagcaaacaaaca
acorn worm  gcctttaatgttctctgggcctttgtacgttcacaatgtgggcacact---agagaatgacgtgtctcggataaaaacacacttttcaatt-atctagttatagtgtagg-ctgtgaagcaaacaaacaatca
sea urchin  -------------------ccattgtcccttgacaatgtatgcacagctcgtgatgatgacgtctgccccataaaaacacacttttccattggtctaggtttcctgtaagcctttgaagtaaaca--------
```

Figure S36: *Ptf1a* enhancer

```
human SIX1     -agacttgtccccctggcatctcaggttaa--agaaggaggtccaggaaaatgt-ggtagagccattacaagaaacccgagccgcagtcatca--------
chimaera six1  tgaggtcggccctccagcttctcgggttaa--aggaggcgggcctgcaaaatgt-ggtagagccattacaagaaacccgagatccattcatcatgcactca
chimaera six2  --ggtcgaccccaccagcctctcgggttaggtagtgggagg-ttgtcaaaatacaagagcagccattagtagaaacgtgatatctgttcatca--------
lancelet       -agggtcggccctctgccggctcgggttgt--agggggagg-tttagaaaatat-ggtatagtcattaagcgaaatttgatacccattcatcatgcgc---
acorn worm     tcaggtcagccctctcgcgtctcgggttga--agtaggagg-tttaagaaatat-ggcatagtcattaagggtaattcgatacccattcacgatctagtca
```

Figure S37: *Six1/2* enhancer

```
human SP8     gaggccgcagattggctggcc--gcgcctccgcggcacttcaaagccggagagggagctacaattg--tggtggaat-gggcggaactgatcatt-gtattgcacacct
chimaera sp8  -----ccctgattggctgccg--gggtctgagcggtacttcaaaggcgagagaggcgctacaattg--cggttgaatggggcggaactgatcatt-gtgttgcacacct
chimaera sp9  -------ctgattggccgagggctcggcacagcggcacttcaaagcccaca--cccgctacaatta--gggtcgagt------------------------------cg
lancelet      gagctcgctgattggtcgagag-gggcctgagcgggccttcaaagggaagc--cacaccacaattatatggtcaaat-gggcggaaccgaccatttgtattgtacacct
acorn worm    --------tcattggtggatca-gccgataggcggctcttcaaagcccttt-gcaagcggcaattatcgggccaaat-gggcggacctggtcatttgcat---------
```

Figure S38: *Sp8/9* promoter

```
human ZFP36L1    gcttgt---------------------------cactgcacat-caatataaaaaa---gcttatttaacttatcaaaacgtattt-attgccaaactatgcttt-----tttttgttaattttgt------tcatattt
human ZFP36L2    tttttagctgttttgagttgattcgcaccactgcaccacaact-caatatgaa------aactatttaactta------tttatta---tcttgtgaaaagtata------caatgaaaattttgt-tcatactgtattt
chimaera zfp36l1 gcttct---------------------------cactgcacacacaacacaaaaaaattgcttatttaacttatc-gaacatattt-attgccaaac--------------atatgcattttttgt------ttgtattt
chimaera zfp36l2 tttttagctgttgagtttttgatggcccactggtggcccagct-cgctataa-------aactatttaactta------tttatta---tctcg-agaaagtata------cattggtaa-tttgt-tcatactgtattt
lancelet         tattta-----------------------------------------------------acttatgtgactta------tttatttgaaccttatacgtaacgttatgcatttttggtaaatttattatgctcgttatgt
acorn worm       --------------------------------------------------------------tatttaagttaacaggccttattt---atttgaagtaagcatt------ttttggtaaatttataatgcctcctattt

human ZFP36L1    atcgggatgacaaat---ccataga-atatatt---cttttatg-ttaaattatgatcttca-tattaatcttaaaattttgtgacgtgtctttttcct-----------------------------------------
human ZFP36L2    atcaagtatgatgaaaagcaatagatatatattcttttattatg-ttaaattatgattgccattattaatcggcaaaatgtggagtgtatgttcttttcacagtaatatatgcc--------------------ttttgt
chimaera zfp36l1 atcaaaaatgatgaa---ccataga-gtatatt-ttcttttatgtttaaattatgatcttc----ttaatcttaagattatgaggtcctttttttggtc-----------------------------------------
chimaera zfp36l2 atcgcgtatgatgaaaa-caatagatatatattcttttattatgtttaaattatgattgccattattaatctgcagaatgtggagtgtatgttgttttcacagtaatatatgccttttttgttgggtttttttgttttgt
lancelet         acgaga--cgatgagag-tgatagatatttatt---atattatgtttaatttatgattgtt--tgtgaatcacaggattggagtgtgtgtactatgtgtatggtaatatgtg----------------------------
acorn worm       atcgggttccatgtggg-acatagatatatatt-ataaactttg-ttaaattatgattgtgaattgaaatggacagagactggagtgt----------------------------------------------------

human ZFP36L1    ----------------tttttt
human ZFP36L2    aacttcac-ttggttattttat
chimaera zfp36l1 ----------------tttttt
chimaera zfp36l2 aacttcactttggttattttat
lancelet         ----------------------
acorn worm       ----------------------
```

Figure S39: *Zfp36l* 3′-UTR

```
human EBF1      gggcccggtatggatgattaggtt------tcgggctttgaattgttgacaaggcaggggtaatgaattgcgggacactggctcctgcatttccccctcattagtcagtattagctacattaatagtaataatt------
human EBF3      -------------------------------------------------------------------------------------cacatgtgcccctcattaagctgcattacctgcattaaaactaataattgccact
chimaera ebf1   ggcccagccgaggatgattagctttgtggatttgactgtgatttgttgacaaggcagagg-gatgaattgtgggac--ttgagcaagtgtgtgactctcattattcagtattagccgcattaatagtaataatt------
chimaera ebf3   ------------------------------------------------------------------------------ttgcacacatgtgcccctctcattaagctgcattacctgcattaaaactaataattgccact
lancelet        ----------------------------------------------------------------------------------ttgcaggcatgcctctcattaagttacatcacatgcattagaagtaatcattcctgct

human EBF1      ---------ggatatattcatcatttaaaatgttttaataaatgtttggacagtacccgatcagagctgtcaaa-------
human EBF3      cagagctgtgcgcccattaatcatttataatgttttaataagtttttaatcaggacctaa--aaggctagagagccgcata
chimaera ebf1   ---------acatgaattaatcatttataatgttttaataaatgtttggtcagcacctgatctacactgtcaaa-------
chimaera ebf3   cagctctgtgctcacattaatcatttataatgttttaataagtttttaatcaggatttga--aaggctagaaagct-----
lancelet        cacctccaagcccccattaatgatttaccaggtcctaatcaggttttgatgggagtgtac--ggggctagcgagctgtgca
```

Figure S40: *Coe* intron element 2

```
human IRX1      ---ttttctacttgaacatgaaataatgattttttgctcatataattacaaagctaacatctgatagagtcagcatccaggggggattatcgcatgcatgagtattagacctcattaccagcttgactgcaaaattatta
human IRX3      tcattttctatttgaacatgaaataatgattttctgcccgtctaattacaaagccaacatctgatcaagccagcatccagaggggattatcgcatgcacgagtattagacctcattaccagcttgagtgcaaaattatta
human IRX4      tcatttttctgctgaacatgaaataatgattttctgccagtataattacaaagctaacatctgatagagtcagcatccaggggggattatcacaggcatgagtattagacctcattaccagcttgactgcaaaattatta
human IRX6      ----ttttttcctgaacatgaaataatgattttctgcccgtctaattacaaagccaacatctgatcaagccagcatccagaggggattatcgcatgcacgagtattagacctcattaccagcttgagtgcaaaattatta
chimaera irx1   ----tttctacttgaacatgaaataatgattttttgctcatataattacaaagctaacatctgatagagtcagcatccaggggggattatcacatgcatgagtattagacctcattaccagctcgactgcaaaattatta
chimaera irx3   tcattttctatttgaacatgaaataatgattttctgccagtctaattacaaagccaacatctgatcaagccggcatccagaggggattatcacatgcacgagtattagacctcattaccagcttgagtgcaaaattatta
chimaera irx4   ---tttttttgctgaacatgaaataatgattttctgccagtataattacaaagctaacatctgatagagtcagcatccaggggggattatcacatgcatgagtattagacctcattaccagcttgactgcaaaattatta
chimaera irx6   ----ttttttcctgaacatgaaataatgattttctgccagtctaattacaaagccaacatctgatcaagccggcatccagaggggattatcacatgcacgagtattagacctcattaccagcttgagtgcaaaattatta
lancelet IrxA   ----------------------------------------------------------------------------------------------------agaattataactaattataagcatgactgccacaatatta
lancelet IrxC   ----------------------------------------------------------------------------------------------------agaattataactaattgtaagcatgactgccacaatatta

human IRX1      catcttgtaattggatggtgag--------atttatatacagattgg----ccgggtt-tctgtaagattgtaattacaagcttcattggtttgctacttatcgga-acttcacagaga----aaacaactggtaaaatg
human IRX3      catcttgtaattggatggtgag--------atttatatacagatcgg----cccggtt-tctgtaagattgtaattacaagcttcgttggttagctacttatcagaactttgcaggaaaaca-aaacaaatgactgagga
human IRX4      catcttgtaattggagggtgag--------atttatatacagattgg----ccgggtt-tctgtaagattgtaattacaagcttcgttggtttgctacttatcaca-cggtgcaggaggcggc--aaaaacaagc-agag
human IRX6      catcttgtaattggatggtgag--------atttatatacagatcgg----cccggtt-tctgtaagattgtaattacaagcttcgttggttagctacttatcact-cagcacagaagag---aggaaaataagc-aaca
chimaera irx1   catcttgtaattggatggcgag--------atttatatacagattgg----ccaggtt-tctgtaagattgtaattacaagcttcattggtttgctacttatcgga-acttcacagaga----aaacaactggtaaaatg
chimaera irx3   catcttgtaattggatggtgag--------atttatatacagatcgg----cctggtt-tctgtaagattgtaattacaagcttcgttggttagctacttatcagaactttgcaggaaaaca-aaacaaatgactgagca
chimaera irx4   catcttgtaattggatggcgag--------atttatatacagattgg----cccggttctctgtaagattgtaattacaagcttcgttggtttgctacttatcaca-cagtgcaggaggaggcaaaaaaacaagc-agag
chimaera irx6   catcttgtaattggatggtgag--------atttatatacagatcgg----cctggtt-tctgtaagattgtaattacaagcttcgttggttagctacttatcaca-cagcacaggaga----aaaaaaataagc-aaca
lancelet IrxA   caactgataattggaaggttggatggcttcatttgcatgtagagctgagacctgggtt-cccaccaga---caattatagctttggttagagggagcctcttggct-ccatgcaggatgctgctaca---------cacc
lancelet IrxC   catctgataattggaaggttggatggcttcatttgcatgtagagctgagacctgggtt-cccaccaga---caattatagctttggttagagggagcctcttggct-caatgcaggatgctgctaca---------cacc

human IRX1      aaatgagcatttcattaacctattgccttatcatgggaaaacctgtgtaacagccgtgtatgcattaacataaatag---------
human IRX3      aaacgagcatttcattaacctgtaacccgagcgcgaggg-----------------------------------------------
human IRX4      ggaaggccatttcattaagctgtaaccctatcaagggaggagcgctgtaacgtccctgtatttattaggccaaacag---------
human IRX6      gaaaaggcatttcattaacccataaccctatcaagggaaagccactgtaacacccatgtctgcattagggagaacagctaaattca
chimaera irx1   aaatgagcatttcattaacctatcgccttatcatgggaaaacctgtgtaacgttcatgtatgcattaacataaatag---------
chimaera irx3   aaatgagcatttcattaacctgtaaccctatcatgggggaacctgtgtaacattcatgtatgcattaaggaaaatgggttcatcca
chimaera irx4   gaaagaccatttcattaacctgtaaccctatcaagggaggagcactgtaacatccctgtatttattaggccaaacag---------
chimaera irx6   gaaagagcatttcattaacccgtaaccctatcaagggagaactgctgtaacatccatgtttgcattagggagaacag---------
lancelet IrxA   acaagaccactttattaacc------------------------------------------------------------------
lancelet IrxC   acaagaccactttattaacc------------------------------------------------------------------
```

Figure S41: *Irx* enhancer

```
human NR2F1     ----------------------aaacaagaattaggggaaaataacattttccaaataattataaaaaattgtcctgtgtctatgtatctatatctg---ttttgta-tttttttctggttccaaaccagatt-tcctgt
human NR2F2     -----------------------------------------------ttttccaaattatt------aattgtcctgtgtctatgtacctctagctgttcttttttg-tacttttctggttccaaaccagtttattctgt
chimaera nr2f1  gcgcatttaaaagacaga---atcaagagattagggggaaaataacattttccaaataattat---aaattgtcctgtgtctatgtatctatatctg---ttttgtattttttttctggttcaaaaccaggtt-tcgtgt
chimaera nr2f2  gtgcatttaaacaagaatttcaaaattaggaggaaagaaaagtaacattttccaaattattat---aaattgtcctgtgtctatgtatctatagctg---ttttgta-ttttttcctggttccaaaccagtctcctctgt
lancelet        ---------------------------------------------------------------------------tgtgtctatgtatctt--gctg---ttttata-----tacatggttccaaaccaaacg-ttaagt

human NR2F1     gatt-ctatactaataatttttgatataaccctttgcttcttataatgagtgcgatatatgttgtcgaggc--tgttcttcaagaattaaaattgaagtga----aaatttaaacaaaaa
human NR2F2     ggtt-ctataata--ag-ttttgatataatc-ttggcttctt-----aaaaactgtgtatcattaaaatat-atgttctgcaagaattaaaactgagtccatgaaaataccataggaaga
chimaera nr2f1  gattcctatactaataatttttgatataaccttttgcttcttataatgagtgagatttatgtcgtcaaagctatgttctccaagaattaaaattgaaatca-------------------
chimaera nr2f2  gatt-ctctactaataa-ttttgatataacc-attgcttcttat--cagatacggtgtatcacgtaaat---atgttctgcaagaattaaaactgaattcactaaaatgttagataaaaa
lancelet        gatt-tactaataataa-ttttgatataacc-----------------------------------------------------------------------------------------
```

Figure S42: *Nr2f* 3′-UTR
